# Synthesis of Antiviral Perfluoroalkyl Derivatives of Teicoplanin and Vancomycin

**DOI:** 10.1002/cmdc.202000260

**Published:** 2020-07-30

**Authors:** Ilona Bereczki, Magdolna Csávás, Zsolt Szűcs, Erzsébet Rőth, Gyula Batta, Eszter Ostorházi, Lieve Naesens, Anikó Borbás, Pál Herczegh

**Affiliations:** ^1^ Department of Pharmaceutical Chemistry University of Debrecen Egyetem tér 1 4032 Debrecen Hungary; ^2^ Doctoral School of Pharmaceutical Sciences University of Debrecen Egyetem tér 1 4032 Debrecen Hungary; ^3^ Department of Organic Chemistry University of Debrecen Egyetem tér 1 4032 Debrecen Hungary; ^4^ Department of Medical Microbiology Semmelweis University Mária u. 41 1085 Budapest Hungary; ^5^ Rega Institute for Medical Research KU Leuven 3000 Leuven Belgium

**Keywords:** antiviral, coronavirus, glycopeptide antibiotic, influenza virus, perfluoroalkyl

## Abstract

The limited scope of antiviral drugs and increasing problem of antiviral drug resistance represent a global health threat. Glycopeptide antibiotics and their lipophilic derivatives have emerged as relevant inhibitors of diverse viruses. Herein, we describe a new strategy for the synthesis of dual hydrophobic and lipophobic derivatives of glycopeptides to produce selective antiviral agents without membrane‐disrupting activity. Perfluorobutyl and perfluorooctyl moieties were attached through linkers of different length to azido derivatives of vancomycin aglycone and teicoplanin pseudoaglycone, and the new derivatives were evaluated against a diverse panel of viruses. The teicoplanin derivatives displayed strong anti‐influenza virus activity at nontoxic concentrations. Some of the perfluoroalkylated glycopeptides were also active against a few other viruses such as herpes simplex virus or coronavirus. These data encourage further exploration of glycopeptide analogues for broad antiviral application.

## Graphical abstract

**Plan F**: Perfluoroalkylated blocks were incorporated into azido glycopeptide derivatives of vancomycin and teicoplanin. The teicoplanin derivatives displayed strong antiviral activity against a diverse panel of viruses at nontoxic concentrations. The vancomycin derivatives showed cytotoxicity, while being inactive against influenza viruses. Perfluorobutylated teicoplanins displayed excellent antibacterial activity against a panel of Gram‐positive bacteria.

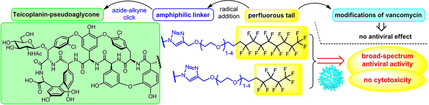

## Introduction

Many biologically active small molecules contain one or more fluorine substituents, owing to the beneficial effect of fluorination on pharmacokinetic and pharmacodynamic properties.^1^, ^2^, ^3^, ^4^, ^5^, ^6^, ^7^ The use of fluorine substituents in medicinal chemistry has continuously increased,^8^, ^9^, ^10^ and currently more than 50 % of blockbuster drugs are fluorinated. In these compounds, the presence of a few fluorine atoms strongly modifies the biological activity and chemical reactivity, while having relatively low impact on the physical properties.^11^, ^12^ On the other hand, the physical characteristics of perfluorinated compounds are very different from those of the hydrogen‐containing analogues. Most importantly, perfluorination simultaneously enhances hydrophobicity and lipophobicity,^13^, ^14^ a unique characteristic that creates exceptional biomedical possibilities. Although the number of pharmacologically applicable molecules containing perfluoroalkyl substituents is currently still limited, polyfluorinated compounds have shown promise in several areas of medicinal chemistry.^15^, ^16^, ^17^

In recent years, we conducted a systematic study on the synthesis of lipophilic derivatives of the glycopeptide antibiotics vancomycin, teicoplanin and ristocetin, and this yielded several new antibiotics with promising antibacterial and antiviral activity.^18^, ^19^, ^20^, ^21^, ^22^ We demonstrated that the high lipophilicity of the side chains (C_8_−C_10_ alkyl groups) in these molecules is essential for antiviral activity against, among others, influenza virus. However, antiviral activity proved accompanied by high cytotoxicity, probably due to a membrane‐disrupting effect of the highly lipophilic side chains.^22^ In addition, we found that cytotoxicity was reduced by decreasing the overall lipophilicity of the compounds following incorporation of a tetra(ethylene glycol) linker between the peptide core and the lipophilic group.^22^ Hence, we assumed that attaching perfluoroalkyl groups that are highly hydro‐ and lipophobic might confer antiviral activity without creating a membrane‐disrupting effect. Namely, it has been shown that triphilic polymers bearing bulky and lipophobic perfluorinated blocks do not mix well with the hydrocarbon chains of lipids, explaining their low membrane partition coefficients.^23^, ^24^ This is the reason why fluorinated surfactants are non‐cytolytic, that is, they are unable to solubilize membranes, as opposed to their hydrogenated counterparts.^25^

Herein, we report on the synthesis and antiviral plus antibacterial evaluation of perfluoroalkyl derivatives of vancomycin aglycone and teicoplanin pseudoaglycone. Although synthetic modification of glycopeptide antibiotics is increasingly recognized in the context of antiviral^26^ and antibacterial^27^ drug design, our study is the first to explore conjugation of glycopeptides with perfluoroalkyl groups.

## Results and Discussion

### Synthesis

**Scheme 1:**
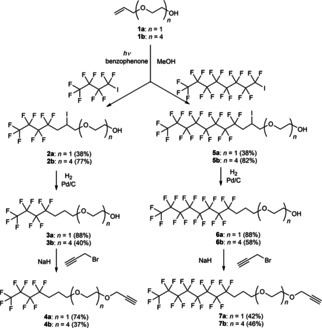
Assembly of the clickable perfluoroalkyl side chains **4 a**, **4 b**, **7 a** and **7 b**.

Our recent results on semisynthetic lipoglycopeptides revealed that the type and length of the linkers significantly modifies the antiviral activity and cytotoxicity.^22^ Herein, we have chosen the **1 a** and **1 b** allyl ethers of ethylene and tetra(ethylene glycol)s as linker chains between perfluoroalkyl substituents and the antibiotic molecules (Scheme 1). A light‐promoted atom‐transfer radical addition reaction^28^ of the commercially available perfluorobutyl iodide and perfluorooctyl iodide onto the double bond of **1 a** and **1 b** resulted in **2 a** and **2 b**, as well as **5 a** and **5 b** in good yields. Attempted reductive removal of iodo substituent with LiAlH_4_ proceeded with low efficacy. Fortunately, catalytic hydrogenation of the iodo derivatives gave **3 a** and **3 b**, as well as **6 a** and **6 b** with moderate to high yields. Finally, these derivatives were reacted with propargyl bromide, respectively, to produce propargylated compounds **4 a** and **4 b** and **7 a** and **7 b** that are suitable for the azide alkyne cycloaddition click reaction.

**Scheme 2:**
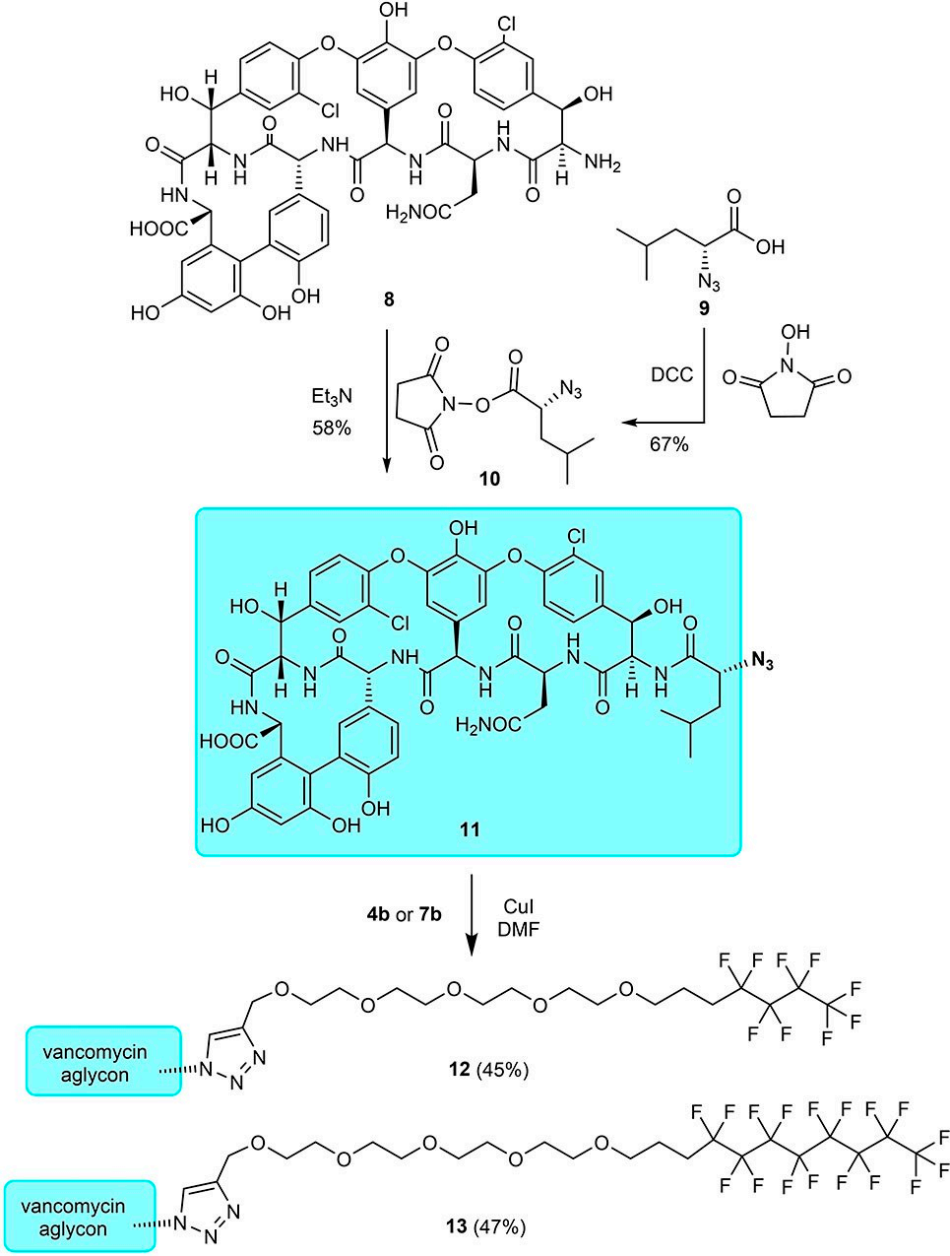
Synthesis of vancomycin aglycone azide **11** and its conjugation with fluorous side chains.

For the derivatization of vancomycin aglycon, an N‐terminal azido analogue **11** was prepared (Scheme 2). Vancomycin hexapeptide **8** obtained from vancomycin aglycone by Edman degradation^29^ was acylated with d‐azidoleucine succinimide ester **10** prepared from d‐azidoleucine **9**.^30^ A copper(I)‐catalyzed 1,3‐dipolar cycloaddition reaction of **4 b** or **7 b** with **11** resulted in perfluorobutyl and perfluorooctyl derivatives **12** and **13** of vancomycin aglycone.

**Scheme 3:**
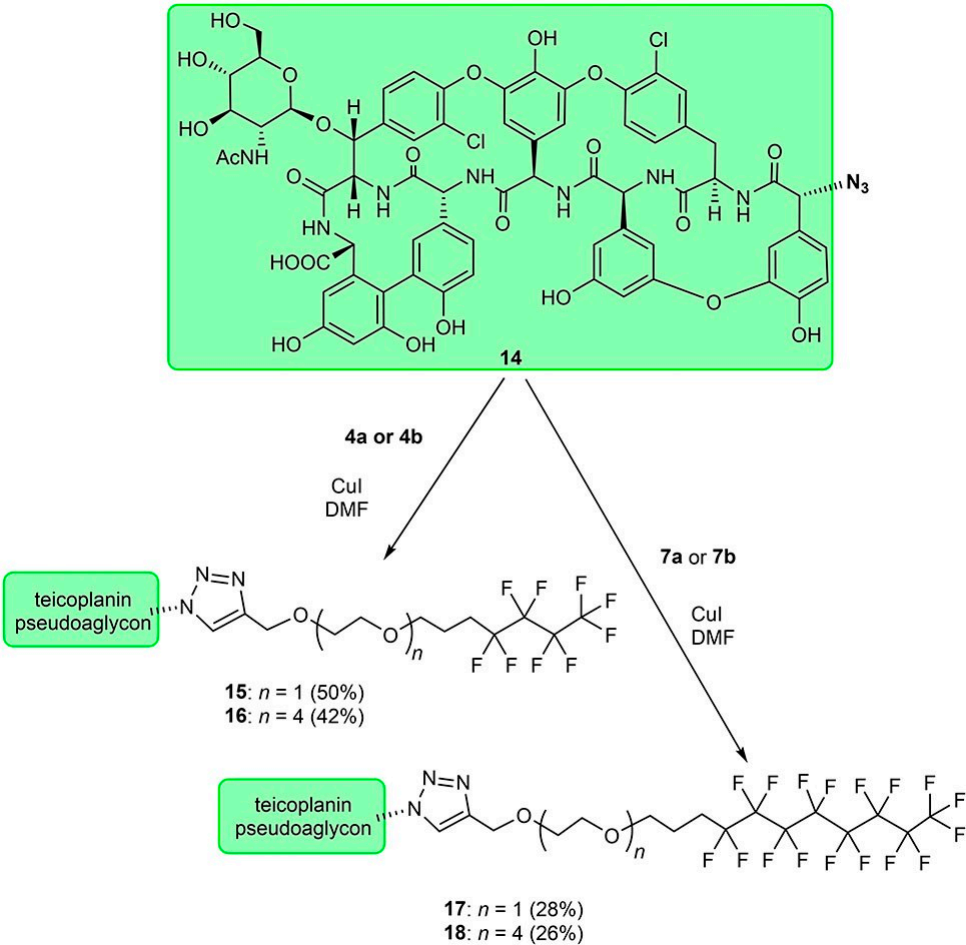
Attachment of fluorous side chains of different length to teicoplanin pseudoaglycone.

Similar click reactions of azido derivative of teicoplanin pseudoaglycone **14** 18 with **4 a**, **4 b**, **7 a**, or **7 b** afforded perfluoroalkyl derivatives **15**, **16**, **17** and **18** with linker chains of different length (Scheme 3).

### Structure elucidation and testing oligomerization

In addition to NMR structure validation (see the Supporting Information, including ^13^C HSQC, HMBC, ^13^C and ^19^F spectra and spectral assignments) we tested compound **17** for possible oligomerization. Strong head to tail dimers are commonly found of glycopeptide antibiotics in aqueous solutions, that is co‐operatively enhanced by ligand binding.^31^, ^32^, ^33^ As **17** is not soluble in water, it was dissolved in MeOD for NMR diffusion experiments.^34^ We used the internal mass standard method as described earlier.^35^ TMS (tetramethylsilane, MW=88.2) and β‐cyclodextrin (β‐CD, MW=1135) were used alternatively. About 20 mg of sample **17** was dissolved in 500 μL MeOD and measured at 300 K temperature. Spectra were recorded with 32 or 64 linear gradient steps and evaluated with topspin 2.1 software using inverse Laplace transformation to obtain diffusion domain. These experiments yielded MW between 7–8 kDa when referenced to internal TMS. (Figure S1). However, in repeated measurements with ∼3 mg of **17** dissolved in MeOD and referenced to internal β‐CD, DOSY yielded only 2.4 kDa mass for **17**, that is closer to monomeric state (Figure S2). In control DOSY experiments of teicoplanin and vancomycin dissolved in [D_6_]DMSO with β‐CD mass reference, DOSY yielded the monomeric molecular masses as expected. Hence, we do not suppose the presence of oligomers in case of dilute MeOD solutions of **17**.

### Biological evaluation

Vancomycin aglycone derivatives **12** and **13** were inactive against various influenza strains and both were highly cytotoxic in MDCK cells (Table 1). Perfluorobutyl and perfluorooctyl derivatives **15**, **16** and **17** of teicoplanin pseudoaglycone displayed robust activity against influenza A and B viruses with a favorable selectivity (i. e., ratio between cytotoxicity and activity). In contrast, the similar perfluorooctyl derivative **18** with a tetra(ethylene glycol) linker displayed high cytotoxicity without antiviral activity. Although this marked difference between vancomycin and teicoplanin derivatives may seem surprising, we previously demonstrated that even a slight difference in the peptide core can lead to very different antiviral properties.^36^

**Table 1:** Cytotoxicity and anti‐influenza virus activity in MDCK^[a]^ cell cultures.

Compound	Cytotoxicity [μM]		Antiviral EC_50_^[d]^ [μM]					
			A/H1N1		A/H3N2		Influenza B	
	MCC^[b]^	CC_50_^[c]^	CPE	MTS	CPE	MTS	CPE	MTS
12	42	4	>100	>100	>100*	>100*	>100	>100
13	0.8	0.8	>100	>100	>100*	>100*	>100	>100
15	100	>100	7.7	7.2	2.3	1.9	8.9	11
16	100	97	5.6	1.6	2.3*	2.5*	5.6	6.5
17	100	44	6.8	8.6	1.2	1.6	4.0	3.4
18	–	4.2	–	>100	–	>100*	–	>100
teicoplanin	>100	>100	>100	>100	>100	>100	>100	>100
14	>100	>100	>100	>100	>100	>100	>100	>100
vancomycin⋅HCl	>100	>100	>100	>100	>100	>100	>100	>100
zanamivir	>100	>100	1.5	3.1	20	9.0	2	1.7

[a] Madin Darby canine kidney cells. Virus strains: A/H1 N1: A/Ned/378/05; A/H3 N2: A/HK/7/87* or A/Victoria/361/11; influenza B virus: B/Ned/537/05. [b] Minimum cytotoxic concentration, i. e., minimal compound concentration causing a microscopically detectable alteration in cell morphology. [c] 50 % cytotoxic concentration based on the formazan‐based MTS cell viability assay. [d] 50 % effective concentration, or concentration producing 50 % inhibition of virus‐induced cytopathic effect, as determined by visual CPE scoring (left column), or by measuring cell viability with the MTS assay (right column).

Besides, activity was noted against herpes simplex virus and vaccinia virus (compounds **15**, **16**, **17** and **18**), adenovirus (**15** and **17**) and coronavirus (**17** and **18**; Table 2). Two other sensitive viruses were respiratory syncytial virus (EC_50_ values: 9.9 μM for **15**; 11 μM for **16** and 4.6 μM for **17**; MTS‐based CPE assay in HeLa cells) and Zika virus (EC50 for **15**: 10 μM; MTS‐based CPE assay in Vero cells; Tables S1 and S2).

**Table 2:** Cytotoxicity and antiviral activity in HEL^[a]^ cell cultures.

Compound	Cytotoxicity	Antiviral EC_50_^[c]^ [μM]					
	CC_50_^[b]^ [μM]	HSV‐1	HSV‐1/TK^−^	HSV‐2	Vaccinia	AdV‐2	HCoV
15	>100	54	39	2.0	10	42	>100
16	>100	33	41	25	48	>100	>100
17	>100	16	16	2.2	7.9	60	40
18	>100	9.8	19	14	17	>100	4.9
cidofovir	>250	2.4	4.7	1.0	10	6.4	–
acyclovir	>250	2.4	146	0.05	>250	–	–
alovudine	>250	–	–	–	–	5.9	–

[a] HEL: human embryonic lung fibroblast cells. Viruses: herpes simplex virus type 1 (HSV‐1) or type 2 (HSV‐2); a thymidine‐kinase deficient (TK^−^) mutant of HSV‐1; vaccinia virus; human adenovirus type 2 (AdV‐2) and human coronavirus (HCoV) 229E. [b] 50 % Cytotoxic concentration based on the formazan‐based MTS cell viability assay. [c] 50 % Effective concentration, based on measuring cell viability with the MTS assay.

The broad activity of teicoplanin pseudoaglycone derivatives **15**, **16** and **17** against different viruses is consistent with our hypothesis that these derivatives may act by disrupting the viral endocytosis process, similarly to what we reported for a glycopeptide active against influenza virus.^37^ Recently, it has been described that glycopeptide antibiotics like teicoplanin, dalbavancin, oritavancin and telavancin are able to prevent the host cell entry processes of Ebola virus, Middle East respiratory syndrome coronavirus (MERS‐CoV) and severe acute respiratory syndrome coronavirus (SARS‐CoV), which results also fit with our surmise.^38^, ^39^

Finally, the antibacterial activity was evaluated on a panel of Gram‐positive bacteria (Table 3). Vancomycin derivatives **12** and **13** displayed moderate activity. Perfluorooctyl derivatives **17** and **18** of teicoplanin pseudoaglycone were inactive, but perfluorobutyl compounds **15** and **16** had excellent antibacterial activity against sensitive and resistant staphylococci and good activity against resistant enterococci having vanA and vanB genes.

**Table 3:** Antibacterial effects.

			MIC^[g]^ [μg/mL]					
	TEI	VAN	**12**	**13**	**15**	**16**	**17**	**18**
Bacillus subtilis ATCC^[a]^ 6633	0.5	0.5	32	16	0.5	2	128	4
Staphylococcus aureus MSSA^[b]^ ATCC 29213	0.5	0.5	4	8	0.5	0.5	16	8
S. aureus MRSA^[c]^ ATCC 33591	0.5	0.5	8	8	0.5	0.5	16	4
Staphylococcus epidermidis ATCC 35984 biofilm	2	2	8	16	0.5	0.5	16	4
S. epidermidis mecA^[d]^	16	4	8	8	0.5	0.5	256	8
Enterococcus faecalis ATCC 29212	2	1	8	4	0.5	1	256	16
E. faecalis 15376 VanA^[e]^	256	256	16	256	1	2	256	32
E. faecalis ATCC 51299 VanB^[f]^	4	128	16	32	4	1	256	32

[a] American Type Culture Collection. [b] Methicillin‐sensitive *S. aureus*. [c] Methicillin‐resistant *S. aureus*. [d] mecA gene expression in *Staphylococcus*. [e] *vanA* gene positive. [f] *vanB* gene positive. [g] Minimum inhibitory concentration. TEI: teicoplanin, VAN: vancomycin.

## Conclusion

We have designed and synthesized the first members of fluoroglycopeptides, a novel type of glycopeptide antibiotic derivatives bearing perfluoroalkyl side chains. Such substituents are not only hydrophobic, but at the same time lipophobic endowing the antibiotic molecules with unique physicochemical properties that are worth exploring and exploiting from an antimicrobial point of view. As the lipophobic perfluoroalkyl blocks are known to have no membrane activity, low cytotoxicity of the perfluoroalkylated glycopeptides were expected. However, the vancomycin derivatives and one of the teicoplanin molecules showed cytotoxicity on canine kidney cells whereas they were inactive against influenza viruses. At the same time, three of the new teicoplanin derivatives displayed high activity against the influenza strains studied, two were active against human corona and adenoviruses, and all teicoplanins proved to be active against vaccinia and herpes viruses without showing cytotoxicity. Moreover, perfluorobutyl derivatives of teicoplanin pseudoaglycone displayed excellent antibacterial activity against a panel of Gram‐positive bacteria.

We hope that these results can open a new way in finding more effective antivirals based on glycopeptide antibiotics.

## Experimental Section

### General information

Compounds **1 b**,^40^ **8**,^29^ **9** 30 and **14** 18 were synthesized according to the literature. Compound **1 a** was purchased from Sigma‐Aldrich, nonafluoro‐1‐iodobutane from TCI and heptadecafluoro‐1‐iodooctane from Alfa Aesar. TLC was performed on Kieselgel 60 F_254_ (Merck) with detection either by immersing into ammonium molybdate‐sulfuric acid solution followed by heating or by using Pauly's reagent for detection. Flash column chromatography was performed using Silica gel 60 (Merck 0.040–0.063 mm). The photoinitiated reactions were carried out in a borosilicate vessel by irradiation with a Hg lamp giving maximum emission at 365 nm. The ^1^H NMR (500, 400 and 360 MHz) ^13^C NMR (125, 100 and 90 MHz) and 2D NMR spectra were recorded with a Bruker DRX‐360, Bruker DRX‐400 and Bruker Avance II 500 spectrometer at 298 or 300 K. For DOSY (Diffusion Ordered Spectroscopy) experiments, Bruker AVANCE‐II, 500 MHz spectrometer was applied, with manufacturer‘s “ledbpgp2 s” pulse sequence. Chemical shifts are referenced to Me_4_Si (0.00 ppm for ^1^H) and to the solvent residual signals. MALDI‐TOF MS analyses of the compounds were carried out in the positive reflectron mode (20 kV) using a BIFLEX III mass spectrometer (Bruker) equipped with delayed‐ion extraction. A nitrogen laser (337 nm, 3 ns pulse width, 106–107 W/cm^2^) operating at 4 Hz was applied to produce laser desorption. 2,5‐Dihydroxybenzoic acid (DHB) was used as matrix and F_3_CCOONa as cationizing agent in DMF. ESI‐QTOF MS measurements were carried out on a maXis II UHR ESI‐QTOF MS instrument (Bruker), in positive ionization mode. The following parameters were applied for the electrospray ion source: capillary voltage: 3.5 kV; end plate offset: 500 V; nebulizer pressure: 0.8 bar; dry gas temperature: 200 °C and dry gas flow rate: 4.5 L/min. Constant background correction was applied for each spectrum, the background was recorded before each sample by injecting the blank sample matrix (solvent). Na‐formate calibrant was injected after each sample, which enabled internal calibration during data evaluation. Mass spectra were recorded by otofControl version 4.1 (build: 3.5, Bruker) and processed by Compass DataAnalysis version 4.4 (build: 200.55.4). The antibacterial evaluations were carried out as it was described in our previous publication.27b

**Table 4:** NMR data of vancomycin derivatives.

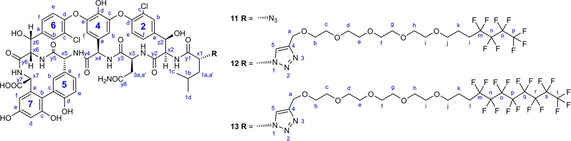						
	**11**		**12**		**13**	
	^1^H	^13^C	^1^H	^13^C	^1^H	
y1	–	177.2113	–	173.99	–	174.55
y8 (Asp)	–	173.9445	–	171.2	–	171.52
y2	–	172.0452	–	169.9	–	170.39
y3	–	171.4567	–	169.09	–	169.55
y4	–	171.3606	–	169.02	–	168.79
y5	–	169.1608	–	168.25	–	166.96
y7	–	n.d.	–	166.35	–	157.41
y6	–	n.d.	–	166.27	–	155.81
7e	–	157.5295	–	157.03	–	155.15
7c	–	156.6308	–	155.36	–	150.35
5d	–	155.5733	–	154.75	–	148.80
2d	–	151.3203	–	150.02	–	147.79
4e	–	150.7161	–	148.4	–	147.61
4c	–	149.3844	–	147.45	–	147.51
6d	–	148.395	–	147.16	–	143.94
6a	–	141.2641	–	143.63	–	143.94
2a	–	139.7893	–	142.25	–	142.57
7a	–	138.83	–	139.28	–	139.48
5b	7.09	136.83	7.22	135.81	7.23	136.30
4d	–	135.07		133.6	–	133.94
4a	–	129.33		128.42	–	128.85
2b	7.2	129.91	7.42	128.24	7.4	128.84
6b	7.56	128.57	7.89	127.28	7.88	127.74
6f	7.56	128.57	7.46	126.94	7.47	127.45
2c	–	128.77		126.51	–	127.01
2f	7.22	128.18	7.52	126.94	7.48	127.44
5a	–	127.16		125.81	–	126.86
6c	–	126.82		125.64	–	126.19
5f	6.87	127	6.75	124.51	6.72	124.97
2e	n.d.	n.d.	7.23	124.09	7.25	124.50
6e	n.d.	n.d.	7.23	122.74	7.24	123.30
5c	–	123.31		122.36	–	126.08
7b	–	119.11		117.21	–	122.71
5e	6.82	118.44	6.68	115.62	6.69	116.01
4f	5.49	106.59	5.6	106.82	5.57	107.11
7f	6.38	108.63	n.d.	n.d.	6.6	108.20
4b	5.26	105.23	5.21	104.12	5.21	104.49
7d	6.36	103.3	6.27	101.23	6.29	101.63
z6	5.18	72.76	5.18	71.01	5.15	71.35
z2	5.3	72.24	5.12	70.72	5.15	71.06
x6	4.09	63.7	4.17	61.52	4.18	61.90
x1	3.81	61.54	5.73	60.67	5.76	60.97
x2	4.33	60.43	4.73	58.99	4.73	59.45
x7	4.42	55.28	4.34	58.99	4.33	59.29
x4	5.96	55.28	5.7	54.41	5.71	54.73
x5	4.72	52.72	4.48	53.39	4.48	53.41
x3	n.d.	n.d.	4.33	50.75	4.35	51.15
1a,a’	2.36	39.26	1.92/2.04	39.96	1.91, 2.03	40.08
3a,a’	n.d.	n.d.	2.16/2.58	36.26	2.89	35.61
1b	1.75	24.64	1.23	23.96	1.18	24.36
1c	n.d.	n.d.	0.9	21.04	0.82	22.60
1d	n.d.	n.d.	0.83	22.15	0.89	21.30
side chain						
triazole CH (5)			8.18	122.61	8.2	123.13
triazole C (4)			–	118.32	–	118.01
OCH_2_ (a)			4.55	63.15	4.52	63.51
OCH_2_			3.52	69.41	3.5	69.80
OCH_2_			3.54	69.36	3.5	69.72
OCH_2_			3.52	69.11	3.5	69.49
OCH_2_			3.58	68.62	3.56	69.00
			3.5	68.11	3.48	68.50
CH_2_ (l)			2.29	26.45	2.26	26.9
CH_2_ (k)			1.77	19.92	1.75	20.30

n.d.: not determined .

#### 2‐((4,4,5,5,6,6,7,7,7‐Nonafluoro‐2‐iodoheptyl)oxy)ethan‐1‐ol (2 a)

Nonafluoro‐1‐iodobutane (2.1 mL, 4.15 g, 12 mmol) and benzophenone (20 mg, 0.11 mmol) were added to a solution of 2‐(allyloxy)ethan‐1‐ol (**1 a**; 1.02 g, 10 mmol) in methanol (15 mL). Argon gas was bubbled through the solution and then irradiation occurred for 10 min. The solvent was evaporated, and the product was purified by flash column chromatography (hexane/acetone 8 : 2) to yield **2 a** (1.7 g, 38 %) as a colorless liquid. *R*_f_=0.34 (hexane/acetone 7 : 3); ^1^H NMR (400 MHz, CDCl_3_): *δ=*4.44–4.35 (m, 1H, CIH), 3.83–3.59 (m, 6H, 3CH_2_), 3.13–2.94 (m, 1H, CH_2_), 2.84–2.64 (m, 1H, CH_2_), 2.12 ppm (s, 1H, OH); ^13^C NMR (100 MHz, CDCl_3_): *δ*=117.7, 106.7 (4 C, CF_2_ and CF_3_), 75.8, 72.4, 61.8 (3 C, CH_2_), 37.8 (t, 1 C, CH_2_), 14.9 ppm (1 C, CHI); HRMS (ESI): *m/z* calcd for C_9_H_10_F_9_IO_2_+Na^+^: 470.9479 [*M*+Na]^+^; found: 470.9474.

#### 16,16,17,17,18,18,19,19,19‐Nonafluoro‐14‐iodo‐3,6,9,12‐tetraoxanonadecan‐1‐ol (2 b)

Nonafluoro‐1‐iodobutane (0.944 mL, 1.9 g, 5.5 mmol) and benzophenone (10 mg, 0.055 mmol) were added to a solution of 3,6,9,12‐tetraoxapentadec‐14‐en‐1‐ol (**1 b**; 1.17 g, 5 mmol) in methanol (12 mL). Argon gas was bubbled through the solution and then irradiation occurred for 10 min. The solvent was evaporated, and the product was purified by flash column chromatography (hexane/acetone 7 : 3) to yield **2 b** (2.09 g, 77 %) as a colorless liquid. *R*_f_=0.29 (hexane/acetone 7 : 3); ^1^H NMR (360 MHz, CDCl_3_): *δ*=4.41–4.31 (m, 1H, CIH), 3.84–3.56 (m, 18H, 9CH_2_), 3.20–3.01 (m, 1H, CH_2_), 2.93–2.85 (m, 1H, OH), 2.77–2.57 ppm (m, 1H, CH_2_); ^13^C NMR (90 MHz, CDCl_3_): *δ=*76.2, 72.6, 70.7, 70.65, 70.6, 70.5, 70.4, 61.7 (9 C, CH_2_), 37.4 (t, 1 C, CH_2_), 14.5 ppm (1 C, CHI); HRMS (ESI): *m/z* calcd for C_15_H_22_F_9_IO_5_+Na^+^: 603.0266 [*M*+Na]^+^; found: 603.0260.

#### 2‐((4,4,5,5,6,6,7,7,7‐Nonafluoroheptyl)oxy)ethan‐1‐ol (3 a)

To the solution of **2 a** (0.896 g, 2 mmol) in methanol (15 mL) 10 % palladium on activated charcoal (270 mg) and NaHCO_3_ (420 mg, 5 mmol) were added. The reaction mixture was stirred overnight under H_2_ atmosphere, then filtered through Celite, and the solvent was evaporated. The residue was dissolved in dichloromethane (50 mL), and the solution was washed with distilled water (10 mL) two times, dried with anhydrous Na_2_SO_4_, filtered, and the solvent was evaporated in vacuum. The product was purified by flash column chromatography (hexane/acetone 8 : 2) to yield **3 a** (571 mg, 88 %) as a colorless liquid. *R*_f_=0.50 (hexane/acetone 7 : 3); ^1^H NMR (400 MHz, CDCl_3_): *δ*=3.75 (s, 2H, CH_2_), 3.62–3.53 (m, 4H, 2CH_2_), 2.32–2.11 (m, 3H, CH_2_, OH), 1.98–1.86 ppm (m, 2H, CH_2_); ^13^C NMR (100 MHz, CDCl_3_): *δ*=72.2, 69.7, 61.9 (3 C, CH_2_), 27.9 (t, 1 C, CH_2_), 20.9 ppm (1 C, CH_2_).

#### 16,16,17,17,18,18,19,19,19‐Nonafluoro‐3,6,9,12‐tetraoxanonadecan‐1‐ol (3 b)

LiAlH_4_ (304 mg, 8 mmol) was added under argon to a solution of **2 b** (2.32 g, 4 mmol) in abs. THF (20 mL), and the reaction mixture was stirred overnight. Then 10 % Na_2_SO_4_ solution (5 mL) was added, and the mixture was stirred for 30 min. After filtration through Celite, the solvent was evaporated, and the product was purified by flash column chromatography (hexane/acetone 7 : 3) to yield **3 b** (728 mg, 40 %) as a colorless liquid. *R*_f_=0.40 (hexane/acetone 7 : 3); ^1^H NMR (360 MHz, CDCl_3_): *δ*=3.77–3.52 (m, 18H, 9CH_2_), 2.28–2.10 (m, 2H, CH_2_), 1.95–1.83 ppm (m, 2H, CH_2_); ^13^C NMR (90 MHz, CDCl_3_): *δ*=72.7, 70.7, 70.6, 70.4, 70.3, 69.8, 61.8 (9 C, CH_2_), 27.9 (t, 1 C, CH_2_), 20.8 ppm (1 C, CH_2_); HRMS (ESI): *m/z* calcd for C_15_H_23_F_9_O_5_+Na^+^: 477.1299 [*M*+Na]^+^; found: 477.5.

**Table 5:** NMR data of teicoplanin derivatives.

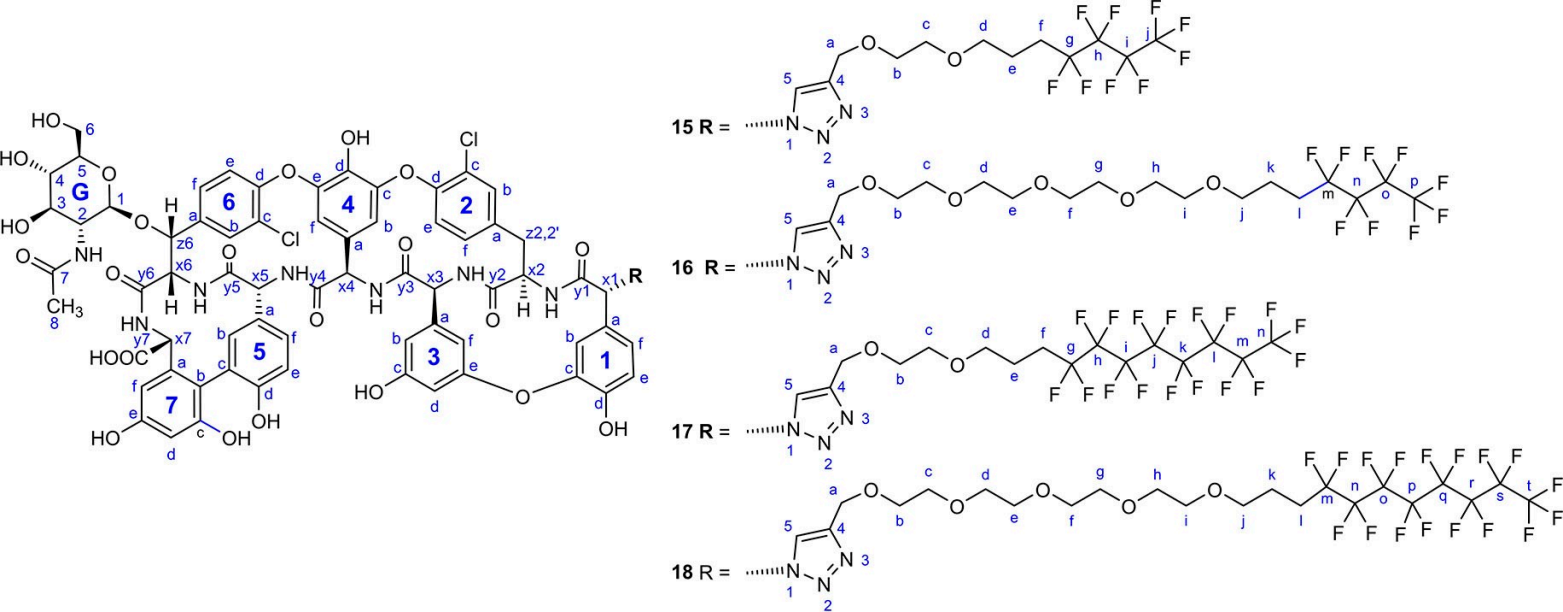								
	**15**		**16**		**17**		**18**	
	^1^H	^13^C	^1^H	^13^C	^1^H	^13^C	^1^H	^13^C
y1	–	174.36	–	173.79	–	n.d.	–	173.47
y7	–	170.02	–	169.90	–	169.67	–	169.94
y4	–	169.68	–	169.44	–	169.67	–	169.37
y2	–	169.36	–	169.25	–	169.17	–	169.19
y5	–	169.00	–	168.87	–	168.58	–	168.83
y3	–	168.33	–	168.22	–	167.16	–	168.17
y6	–	167.46	–	167.30	–	166.53	–	167.30
C=O(NAc) G7	–	165.94	–	165.58	–	n.d.	–	165.59
3c	–	158.56	–	158.46	–	159.90	–	158.45
7e	–	157.75	–	157.65	–	158.51	–	157.60
3e	–	156.87	–	156.75	–	158.24	–	156.73
7c	–	155.75	–	155.62	–	157.05	–	155.67
5d	–	155.61	–	155.49	–	155.95	–	155.44
2d	–	151.16	–	151.07	–	155.95	–	151.04
6d	–	149.21	–	149.10	–	151.15	–	149.08
4c	–	148.96	–	148.83	–	149.53	–	148.77
4e	–	148.02	–	147.93	–	148.13	–	147.91
1d	–	147.49	–	147.42	–	147.60	–	147.40
triazole‐q (4)	–	143.78	–	143.76	–	143.59	–	143.75
6a	–	142.74	–	142.60	–	142.19	–	142.61
1c	–	140.94	–	140.89	–	139.09	–	140.83
3a	–	139.49	–	139.68	–	138.17	–	139.37
7a	–	138.62	–	138.64	–	135.18	–	138.60
5b	7.12	136.1	7.12	136.01	7.17. 7.09	136.3	7.11	135.97
2a	–	135.41	–	135.3	–	135.18	–	135.30
4d	–	134.43	–	134.74	–	n.d.	–	134.31
1a	–	n.d.	–	134.36	–	n.d.	–	n.d.
2b	7.21	131	7.2	130.91	7.15	130.24	7.21	130.94
2f	7.94	130.6	7.89	130.46	7.72	131.25	7.92	130.44
4a	–	127.22	–	127.13	–	126.81	–	128.6
6c	–	126.7	–	126.59	–	126.71	–	128
6f	7.26	127.81	7.28	127.8	7.25	127.74	7.27	128
6b	7.86	128.49	7.87	128.35	7.81	128.29	7.87	128.37
2c	–	125.43	–	125.33	–	n.d.	–	127.13
5a	–	125.43	–	125.33	–	n.d.	–	126.59
5f	6.67	125.12	6.66	125.29	6.65	125.02	6.66	125.32
1f	6.9	125.59	6.9	125.48	7.04	124.33	6.9	125.48
2e	7.19	124.84	7.18	124.72	7.08	127.15	7.19	124.73
6e	6.99	119.0	6.96	118.94	7.24	123	7.27	123.05
triazole‐CH (5)	7.74	123.98	7.71	123.81	7.7	123.88	7.72	123.78
5c	–	122.17	–	122.19	–	122.2	–	122.06
1e	7.26	123.24	7.25	123.06	6.89	118.61	7.05	119.37
7b	–	117.82	–	117.71	–	117.87	–	117.71
1b	7.04	119.49	7.04	119.39	6.94	119.6	6.97	118.91
5e	6.65	116.45	6.64	116.38	6.65	116.32	6.64	116.37
3b	6.34	110.03	6.34	109.97	6.32	109.53	6.34	109.97
4b	5.5	108.05	5.56	107.92	5.54	107.65	5.56	108
7f	6.5	107.86	6.52	107.92	6.53	107.73	6.48	107.68
3d	6.38	105.1	6.37	104.97	6.34	104.17	6.37	104.99
4f	5.1	104.59	5.1	104.46	5.09	104.26	5.1	104.47
3f	6.55	104.02	6.55	103.84	6.57	103.39	6.55	103.85
7d	6.32	101.68	6.3	101.51	6.31	101.52	6.3	101.56
G1	4.38	99.27	4.4	98.73	4.37	99.42	4.42	98.57
G5	3.1	76.79	3.11	76.77	3.09	76.44	3.11	76.77
G3	5.4	76.14	5.45	75.58	5.35	76.2	5.44	75.43
G4	3.39	73.38	3.4	73.4	3.4	72.89	3.41	73.4
z6	3.22	70.02	3.25	69.97	3.26	69.13	3.23	70.05
x6	4.16	60.94	4.15	60.88	4.2	60.46	4.14	60.87
G6	3.62	60.25	3.6	60.29	3.39	60.1	3.6	60.38
x3	5.4	58.31	5.41	58.21	5.36	58.41	5.41	58.17
x1	4.33	59.28	4.33	59.22	4.28	59.08	4.36	58.9
x7	7.12	64.04	7.09	64.03	7.06	64.18	7.1	63.98
G2	3.52	55.8	3.52	55.9	3.59	55.23	3.51	55.98
x4	5.65	54.67	5.63	54.65	5.6	54.27	5.64	54.64
x2	4.87	55.46	4.89	55.37	4.97	55	4.88	55.38
x5	4.37	53.61	4.37	53.53	4.38	53.22	4.36	53.54
z2,2’			n.d.	n.d.			3.32, 2.92	36.18
Side chain								
OCH_2_ (a)	4.45	63.29	4.45	63.25	4.45	63.04	4.45	63.23
OCH_2_	3.48	69.34	3.48	69.32	3.48	69.07	3.5	69.67
OCH_2_	3.51	68.9	3.51	69.08	3.51	68.62	3.5	69.38
OCH_2_	3.44	68.48	3.44	68.5	3.44	68.24	3.46	68.48
							3.5	68.39
CH_2_ (e)	1.73	20.25			1.73	20.26		
CH_2_ (f)	2.24	26.76			2.23	26.87		
CH_2_ (k)			1.76	20.23			1.76	20.24
CH_2_ (l)			2.23	26.86			2.28	26.85

#### 2‐((4,4,5,5,6,6,7,7,8,8,9,9,10,10,11,11,11‐Heptadecafluoro‐2‐iodoundecyl)oxy)ethan‐1‐ol (5 a)

Heptadecafluoro‐1‐iodooctane (3.17 mL, 6.55 g, 12 mmol) and benzophenone (20 mg, 0.11 mmol) were added to a solution of 2‐(allyloxy)ethan‐1‐ol (**1 a**; 1.02 g, 10 mmol) in methanol (15 mL). Argon gas was bubbled through the solution and then irradiation occurred for 10 min. The solvent was evaporated, and the product was purified by flash column chromatography (hexane/acetone 8 : 2) to yield **5 a** (2.07 g, 38 %) as a colorless liquid. *R*_f_=0.43 (hexane/acetone 7 : 3); ^1^H NMR (360 MHz, CDCl_3_): *δ*=4.46–4.33 (m, 1H, CIH), 3.83–3.61 (m, 6H, 3CH_2_), 3.16–2.91 (m, 1H, CH_2_), 2.87–2.62 (m, 1H, CH_2_), 2.28–2,14 ppm (m, 1H, OH); ^13^C NMR (90 MHz, CDCl_3_): *δ*=117.9, 111.2 (CF_2_ and CF_3_), 75.8, 72.4, 61.8 (3 C, CH_2_), 37.9 (t, 1 C, CH_2_), 15.0 ppm (1 C, CHI); HRMS (ESI): *m/z* calcd for C_13_H_10_F_17_IO_2_+Na^+^: 670.9352 [*M*+Na]^+^; found: 670.9346.

#### 16,16,17,17,18,18,19,19,20,20,21,21,22,22,23,23,23‐Heptadecafluoro‐14‐iodo‐3,6,9,12‐tetraoxatricosan‐1‐ol (5 b)

Heptadecafluoro‐1‐iodooctane (1.46 mL, 3.0 g, 5.5 mmol) and benzophenone (10 mg, 0.055 mmol) were added to a solution of 3,6,9,12‐tetraoxapentadec‐14‐en‐1‐ol (**1 b**; 1.17 g, 5 mmol) in methanol (12 mL). Argon gas was bubbled through the solution and then irradiation occurred for 10 min. The solvent was evaporated, and the product was purified by flash column chromatography (hexane/acetone 7 : 3) to yield **5 b** (3.2 g, 82 %) as a colorless liquid. *R*_f_=0.40 (hexane/acetone 7 : 3); ^1^H NMR (360 MHz, CDCl_3_): *δ*=4.42–4.32 (m, 1H, CIH), 3.85–3.56 (m, 18H, 9CH_2_), 3.20–2.91 (m, 2H, CH_2_, OH), 2.78–2.58 ppm (m, 1H, CH_2_); ^13^C NMR (90 MHz, CDCl_3_): *δ*=117.8, 114.3, 111.2, 110.8 (8 C, CF_2_, CF_3_), 76.2, 72.7, 70.7, 70.66, 70.6, 70.5, 70.3, 61.7 (9 C, CH_2_), 37.5 (t, 1 C, CH_2_), 14.5 ppm (1 C, CHI); HRMS (ESI): *m/z* calcd for C_19_H_22_F_17_IO_5_+Na^+^: 803.0138 [*M*+Na]^+^; found: 803.0133.

#### 2‐((4,4,5,5,6,6,7,7,8,8,9,9,10,10,11,11,11‐Heptadecafluoroundecyl)oxy)ethan‐1‐ol (6 a)

To a solution of **5 a** (1.296 g, 2 mmol) in methanol (15 mL) 10 % palladium on activated charcoal (270 mg) and NaHCO_3_ (420 mg, 5 mmol) were added. The reaction mixture was stirred overnight under H_2_ atmosphere, then filtered through Celite, and the solvent was evaporated. The residue was dissolved in dichloromethane (50 mL) and the solution was washed with distilled water (10 mL) two times, dried with anhydrous Na_2_SO_4_, filtered and the solvent was evaporated in vacuum. The product was purified by flash column chromatography (hexane/acetone 8 : 2) to yield **6 a** (922 mg, 88 %) as a colorless liquid. *R*_f_=0.58 (hexane/acetone 7 : 3); ^1^H NMR (400 MHz, CDCl_3_): *δ*=3.78–3.72 (m, 2H, CH_2_), 3.61–3.53 (m, 4H, 2CH_2_), 2.29–2.04 (m, 3H, CH_2_, OH), 1.96–1.86 ppm (m, 2H, CH_2_); ^13^C NMR (100 MHz, CDCl_3_): *δ*=118.7, 115.9, 110.9 (8 C, CF_2_, CF_3_); 72.2, 69.8, 61.9 (3 C, CH_2_), 28.1 (t, 1 C, CH_2_), 20.9 ppm (1 C, CH_2_); HRMS (ESI): *m/z* calcd for C_13_H_11_F_17_O_2_+Na^+^: 545.0385 [*M*+Na]^+^; found: 545.0380.

#### 16,16,17,17,18,18,19,19,20,20,21,21,22,22,23,23,23‐Heptadecafluoro‐3,6,9,12‐tetraoxatricosan‐1‐ol (6 b)

LiAlH_4_ (304 mg, 8 mmol) was added under argon to a solution of **5 b** (3.12 g, 4 mmol) in abs. THF (20 mL), and the reaction mixture was stirred overnight. Then 10 % Na_2_SO_4_ solution (5 mL) was added and the mixture was stirred for 30 min. After filtration through Celite, the solvent was evaporated, and the product was purified by flash column chromatography (hexane/acetone 7 : 3) to yield **6 b** (1.52 g, 58 %) as a colorless liquid. *R*_f_=0.30 (hexane/acetone 7 : 3); ^1^H NMR (360 MHz, CDCl_3_): *δ*=3.76–3.51 (m, 18H, 9CH_2_), 2.28–2.10 (m, 2H, CH_2_), 1.95–1.83 ppm (m, 2H, CH_2_); ^13^C NMR (90 MHz, CDCl_3_): *δ*=121.5, 118.8, 114.3, 110.8, 108.2 (8 C, CF_2_, CF_3_), 72.7, 70.6, 70.58, 70.3, 70.2, 69.7, 61.7 (9 C, CH_2_), 27.9 (t, 1 C, CH_2_), 20.7 ppm (1 C, CH_2_); HRMS (ESI): *m/z* calcd for C_19_H_23_F_17_O_5_+Na^+^: 677.1172 [*M*+Na]^+^; found: 677.1166.

#### General procedure for propargylation (4 a, 4 b, 7 a and 7 b)

NaH (60 % dispersion in mineral oil; 2 mmol) was washed with hexane, abs. THF (10 mL) was added and **3 a**, **3 b**, **6 a** or **6 b** (1 mmol) was dissolved in it. After 30 min of stirring, propargyl bromide (80 % solution in toluene; 1.2 mmol) was added, and the reaction mixture was stirred for 3 h. Then ethyl acetate (5 mL) and methanol (1 mL) were added to the mixture, and it was stirred for 15 min. The solvent was evaporated, the residue was dissolved in dichloromethane and the solution was washed with distilled water (3x15 mL). The organic phase was dried on anhydrous Na_2_SO_4_, then it was filtered and evaporated in vacuum. The product was purified by flash column chromatography (hexane/acetone 8 : 2) to yield **4 a**, **4 b**, **7 a** or **7 b** as yellowish liquids.

**1,1,1,2,2,3,3,4,4‐Nonafluoro‐7‐(2‐(prop‐2‐yn‐1‐yloxy)ethoxy)heptane (4 a)**: Yield 267 mg (74 %); *R*_f_=0.58 (hexane/acetone 7 : 3); ^1^H NMR (400 MHz, CDCl_3_): *δ*=4.21 (d, *J*=2.4 Hz, 2H, CH_2_ propargyl), 3.73–3.67 (m, 2H, CH_2_), 3.66–3.61 (m, 2H, CH_2_), 3.56 (t, *J*=6.1 Hz, 2H, CH_2_), 2.43 (t, *J*=2.4 Hz, 1H, CH propargyl), 2.29–2.12 (m, 2H, CH_2_), 1.95–1.85 ppm (m, 2H, CH_2_); ^13^C NMR (100 MHz, CDCl_3_): *δ*=79.6 (1 C, C_q_ propargyl), 74.6 (1 C, CH propargyl), 70.2, 69.9, 69.2 (3 C, CH_2_), 58.5 (1 C, CH_2_ propargyl) 28.0 (t, 1 C, CH_2_), 20.8 ppm (1 C, CH_2_).

**20,20,21,21,22,22,23,23,23‐Nonafluoro‐4,7,10,13,16‐pentaoxatricos‐1‐yne (4 b)**: Yield 180 mg (37 %); *R*_f_=0.46 (hexane/acetone 7 : 3); ^1^H NMR (400 MHz, CDCl_3_): *δ*=4.20 (d, *J*=2.4 Hz, 2H, CH_2_ propargyl), 3.74–3.57 (m, 16H, CH_2_), 3.55 (t, *J*=6.1 Hz, 2H, CH_2_), 2.43 (t, *J*=2.4 Hz, 1H, CH propargyl), 2.27–2.10 (m, 2H, CH_2_), 1.93–1.83 ppm (m, 2H, CH_2_); ^13^C NMR (100 MHz, CDCl_3_): *δ*=74.6 (1 C, CH propargyl), 70.7, 70.5, 70.3, 69.8, 69.2 (9 C, CH_2_), 58.5 (1 C, CH_2_ propargyl) 27.9 (t, 1 C, CH_2_), 20.8 ppm (1 C, CH_2_); HRMS (ESI): *m/z* calcd for C_18_H_25_F_9_O_5_+Na^+^: 515.1456 [*M*+Na]^+^; found: 515.1451.

**1,1,1,2,2,3,3,4,4,5,5,6,6,7,7,8,8‐Heptadecafluoro‐11‐(2‐(prop‐2‐yn‐1‐yloxy)ethoxy)undecane (7 a)**: Yield 234 mg (42 %); *R*_f_=0.64 (hexane/acetone 7 : 3); ^1^H NMR (400 MHz, CDCl_3_): *δ*=4.21 (d, *J*=2.4 Hz, 2H, CH_2_ propargyl), 3.73–3.67 (m, 2H, CH_2_), 3.66–3.61 (m, 2H, CH_2_), 3.56 (t, *J*=6.1 Hz, 2H, CH_2_), 2.43 (t, *J*=2.4 Hz, 1H, CH propargyl), 2.29–2.12 (m, 2H, CH_2_), 1.95–1.85 ppm (m, 2H, CH_2_); ^13^C NMR (100 MHz, CDCl_3_): *δ*=118.7, 116.2, 111.0 (8 C, CF_2_, CF_3_); 79.7 (1 C, C_q_ propargyl), 74.6 (1 C, CH propargyl), 70.2, 69.9, 69.2 (3 C, CH_2_), 58.6 (1 C, CH_2_ propargyl) 28.1 (t, 1 C, CH_2_), 20.9 ppm (1 C, CH_2_); HRMS (ESI): *m/z* calcd for C_16_H_13_F_17_O_2_+Na^+^: 583.0542 [*M*+Na]^+^; found: 583.0535.

**20,20,21,21,22,22,23,23,24,24,25,25,26,26,27,27,27‐Heptadecafluoro‐4,7,10,13,16‐pentaoxaheptacos‐1‐yne (7 b)**: Yield 318 mg (46 %); *R*_f_=0.42 (hexane/acetone 7 : 3); Compound **7 b** was reacted with the azido glycopeptide derivatives without characterization by NMR; HRMS (ESI): *m/z* calcd for C_22_H_25_F_17_O_5_+Na^+^: 715.1328 [*M*+Na]^+^; found: 715.1323.

#### 2,5‐Dioxopyrrolidin‐1‐yl (R)‐2‐azido‐4‐methylpentanoate (10)

2‐Azido‐4‐methylpentanoic acid (**9**; 1.256 g, 8 mmol) and *N*‐hydroxysuccinimide (1.013 g, 8.8 mmol) were dissolved in abs. dichloromethane (50 mL) and after cooling in an ice bath, dicyclohexylcarbodiimide (1.76 g, 8.5 mmol) was added. The reaction mixture was stirred overnight at room temperature, then filtered through Celite and the solvent was evaporated in vacuum, and co‐evaporated with chloroform (2×50 mL) two times. Then it was dissolved in acetonitrile, filtered through Celite and after evaporation the product was purified by flash column chromatography (hexane/ethyl acetate 8 : 2) to yield **10** (1.1 g, 67 %). *R*_f_= (hexane/ethyl acetate 8 : 2); ^1^H NMR (400 MHz, CDCl_3_): *δ*=4.17 (dd, *J*=8.8, 5.8 Hz, 2H, CH), 2.86 (s, 4H, CH_2_), 1.99–1.77 (m, 3H, CH, CH_2_), 1.01 ppm (dd, *J*=9.5, 6.4 Hz, 6H, CH_3_); ^13^C NMR (100 MHz, CDCl_3_): *δ*=168.7, 166.9 (3 C, C=O), 58.3 (1 C, CH), 39.9 (1 C, CH_2_), 25.6 (2 C, CH_2_) 24.9, 22.7, 21.5 ppm (3 C, CH, CH_3_); MALDI‐TOF MS: [M+Na]^+^=277.204 *m/z*. Calcd (C_10_H_14_N_4_O_4_Na) 277.091 *m/z*.

### Compound 11

To the solution of vancomycin aglycone hexapeptide (**8**; 1.5 g, 1.48 mmol) in abs. *N*,*N*‐dimethylformamide (50 mL) triethylamine (206 μL, 1.48 mmol) and **10** (753 mg, 2.96 mmol) were added. The reaction mixture was stirred for 3 h, the solvent was evaporated, and the product was purified by flash column chromatography (acetonitrile/water 9 : 1) to yield **11** (986 mg, 58 %) as a yellow powder. *R*_f_=0.34 (acetonitrile/water 9 : 1); NMR data can be found in Table 4.; MALDI‐TOF MS: [*M*+Na]^+^=1177.089 *m/z*. Calcd (C_52_H_48_Cl_2_N_10_O_17_Na) 1177.247 *m/z*.

### General procedure for click reaction (12, 13, 15, 16, 17 and 18)

To the solution of azide (**11** or **14**; 0.1 mmol) in *N,N*‐dimethylformamide (2 mL) triethylamine (14 μL, 0.1 mmol), alkyne (**4 a**, **4 b**, **7 a** or **7 b**; 0.12 mmol) and Cu^I^ iodide (10 mg) were added. The reaction mixture was stirred overnight, then the solvent was evaporated and the product was purified by flash column chromatography (toluene/methanol 7 : 3→6 : 4 →1 : 1 for **12** and **13** or acetonitrile/water 9 : 1 for **15**, **16**, **17**, and **18**) to yield **12**, **13**, **15**, **16**, **17** or **18**. After lyophilization all of the products were yellow solid foams.

**12**: Yield 71 mg (45 %); *R*_f_=0.53 (toluene/methanol 6 : 4); NMR data can be found in Table 4; HRMS (ESI): *m/z* calcd for C_70_H_73_Cl_2_F_9_N_10_O_22_+Na^+^: 1669.4032 [*M*+Na]^+^; found: 1669.4020.

**13**: Yield 87 mg (47 %); *R*_f_=0.58 (toluene/methanol 6 : 4); NMR data can be found in Table 4; HRMS (ESI): *m/z* calcd for C_74_H_73_Cl_2_F_17_N_10_O_22_+Na^+^: 1869.3904 [*M*+Na]^+^; found: 1869.3873.

**15**: Yield 90 mg (50 %); *R*_f_=0.49 (acetonitrile/water 85 : 15); NMR data can be found in Table 5; HRMS (ESI): *m/z* calcd for C_78_H_69_Cl_2_F_9_N_10_O_25_+Na^+^: 1809.3566 [*M*+Na]^+^; found: 1809.3549.

**16**: Yield 80 mg (42 %); *R*_f_=0.14 (acetonitrile/water 9 : 1); NMR data can be found in Table 5.; HRMS (ESI): *m/z* calcd for C_84_H_80_Cl_2_F_9_N_10_O_28_Na+Na^+^: 1963,4554 [*M*−H+2Na]^+^; found: 1963.4150.

**17**: Yield 56 mg (28 %); *R*_f_=0.50 (acetonitrile/water 85 : 15); NMR data can be found in Table 5.; HRMS (ESI): *m/z* calcd for C_82_H_67_Cl_2_F_17_N_10_O_25_Na_2_+Na^+^: 2053.3078 [*M*−2H+3Na]^+^; found: 2053.3078.

**18**: Yield 52 mg (26 %); *R*_f_=0.23 (acetonitrile/water 9 : 1); NMR data can be found in Table 5.; HRMS (ESI): *m/z* calcd for C_88_H_81_Cl_2_F_17_N_10_O_28_+Na^+^: 2141.4225 [*M*+Na]^+^; found: 2141.4199.

### Antiviral procedures

The CPE reduction assay for influenza virus was described in full detail in previous publications.^41^ The virus strains were: A/PR/8/34 (A/H1 N1); A/Virginia/ATCC3/2009 (A/H1 N1pdm); A/HK/7/87 (A/H3 N2); B/Ned/537/05; and B/HK/5/72. On day 0, Madin‐Darby canine kidney (MDCK) cells in 96‐well plates were infected with influenza virus at a multiplicity of infection (MOI) of 0.0004 plaque forming units (PFU) per cell. After three days incubation at 35 °C, virus‐induced CPE and compound cytotoxicity were scored by microscopy, after which the data were confirmed by formazan‐based MTS cell viability assay (CellTiter 96® AQueous One Solution Cell Proliferation Assay from Promega). The antiviral effect was expressed as the compound concentration producing 50 % inhibition of the virus‐induced CPE (EC_50_). Compound cytotoxicity was expressed as the compound concentration causing minimal changes in cell morphology (MCC), and 50 % cytotoxic concentration (CC_50_) based on MTS assay.^42^

Inhibitory effect against human coronavirus 229E was determined using a CPE reduction assay in human embryonic lung fibroblast (HEL) 299 cells, described in full detail elsewhere.^43^ We also reported the detailed methodology for the other DNA and RNA viruses in the test panel.^44^ The EC_50_, CC_50_ and MCC values were calculated as described.^42^

## Conflict of interest

The authors declare no conflict of interest.

## Supporting information

As a service to our authors and readers, this journal provides supporting information supplied by the authors. Such materials are peer reviewed and may be re‐organized for online delivery, but are not copy‐edited or typeset. Technical support issues arising from supporting information (other than missing files) should be addressed to the authors.

Supplementary
