# Supplementary material for: Synthesis of Antiviral Perfluoroalkyl Derivatives of Teicoplanin and Vancomycin
Source: ChemMedChem. 2020 Jul 30;15(17):1661–71. doi: 10.1002/cmdc.202000260 (PMC7540527; doi:10.1002/cmdc.202000260)
Supplement: Supplementary file 1 — Supplementary [file CMDC-15-1661-s001.pdf]

ChemMedChem

Supporting Information

Synthesis of Antiviral Perfluoroalkyl Derivatives of Teicoplanin and Vancomycin

Ilona Bereczki, Magdolna Csávás, Zsolt Szűcs, Erzsébet Rőth, Gyula Batta, Eszter Ostorházi, Lieve Naesens, Anikó Borbás,* and Pál Herczegh*

Table of contents

Table S1. Cytotoxicity and antiviral activity in HeLa cell cultures S3

Table S2. Cytotoxicity and antiviral activity in Vero cell cultures... S3

Antiviral procedures S4

References S4

^{1}H-DOSY spectra of compound **17**..S**Hiba! A könyvjelző nem létezik.**

NMR Spectra of the compounds S6

Table S1. Cytotoxicity and antiviral activity in HeLa cell cultures

Compound	Cytotoxicity		Antiviral EC_{50}[c] Vesicular stomatitis virus		Antiviral EC_{50}[c] Coxsackie virus B4		Antiviral EC_{50}[c] Respiratory syncytial virus	
	CC_{50}[a]	MCC[b]	Visual CPE score	MTS	Visual CPE score	MTS	Visual CPE score	MTS
**12**	>100	>100	-	-	-	-	20	10
**13**	>100	≥100	-	-	-	-	9.2	10
**15**	43	-	>100	-	>100	-	9.9	-
**16**	>100	>100	>100	>100	>100	>100	12	8.9
**17**	>100	>100	>100	>100	>100	>100	7.5	6.8
**18**	41	-	>100	-	>100	-	>100	-
**DS-10,000**	>100	-	2.5	-	>100	-	0.1	-
**Ribavirin**	>250	-	87	-	126	-	5	-

[a]50% Cytotoxic concentration, as determined by measuring the cell viability with the colorimetric formazan-based MTS assay.
[b]Minimum compound concentration that causes a microscopically detectable alteration of normal cell morphology.
[c]50% Effective concentration, or concentration producing 50% inhibition of virus-induced cytopathic effect, as determined by visual scoring of the CPE, or by measuring the cell viability with the colorimetric formazan-based MTS assay.

Table S2. Cytotoxicity and antiviral activity in Vero cell cultures

Compound	Cytotoxicity		Antiviral EC_{50}[c] Reovirus-1		Antiviral EC_{50}[c] Sindbis virus		Antiviral EC_{50}[c] Coxsackie virus B4		Antiviral EC_{50}[c] Punta Toro virus		Antiviral EC_{50}[c] Yellow Fever virus		Antiviral EC_{50}[c] Zika virus	
	CC_{50}[a]	MCC[b]	Visual CPE score	MTS	Visual CPE score	MTS	Visual CPE score	MTS	Visual CPE score	MTS	Visual CPE score	MTS	Visual CPE score	MTS
**12**	-	-	-	-	-	-	-	-	-	-	-	-	-	-
**13**	-	-	-	-	-	-	-	-	-	-	-	-	-	-
**15**	>100	>100	>100	>100	82	100	>100	>100	>100	>100	>100	>100	9.4	45
**16**	>100	-	>100	-	>100	-	>100	-	>100	-	>100	-	-	-
**17**	>100	-	>100	-	>100	-	>100	-	>100	-	>100	-	>100	-
**18**	>100	-	>100	-	>100	-	>100	-	>100	-	>100	-	-	-
**DS-10,000**	>100	>100	>100	20	28.3	20	40.6	44.7	29.2	58.5	7.2	20	95.5	100
**Mycophenolic acid**	>100	>100	3.3	4	5.7	4	>100	>100	>100	10.6	>100	1.4	0.8	1.5

[a]50% Cytotoxic concentration, as determined by measuring the cell viability with the colorimetric formazan-based MTS assay.
[b]Minimum compound concentration that causes a microscopically detectable alteration of normal cell morphology.
[c]50% Effective concentration, or concentration producing 50% inhibition of virus-induced cytopathic effect, as determined by visual scoring of the CPE, or by measuring the cell viability with the colorimetric formazan-based MTS assay.

Antiviral procedures

Inhibitory effect against human coronavirus 229E was determined using a CPE reduction assay in human embryonic lung fibroblast (HEL) 299 cells, described in full detail elsewhere.[1] The other virus/cell assays were: in HEL cells: herpes simplex virus type 1 (HSV-1, including a thymidine kinase deficient strain HSV-1/TK^{-}), herpes simplex virus type 2 (HSV-2), vaccinia virus and adenovirus; in human cervix carcinoma HeLa cells: respiratory syncytial virus; and in African green monkey kidney Vero cells: yellow fever virus (17D) and Zika virus (strain MR766). Semiconfluent cell cultures in 96-well plates were inoculated with the virus at a multiplicity of infection of 100 CCID50 (50% cell culture infective dose) per well. Together with the virus, serial dilutions of the compounds were added. The plates were incubated at 37 °C during 3 to 6 days until clear CPE was reached. Microscopic scoring was then performed to determine the antiviral activity [expressed as 50% effective concentration (EC50)] and cytotoxicity [expressed as minimum cytotoxic concentration (MCC)], calculated as reported.[2]

References

1. Ç. B. Apaydin, N. Cesur, A. Stevaert, L. Naesens, Z. Cesur, Synthesis and anti-coronavirus activity of a series of 1-thia-4-azaspiro[4.5]decan-3-one derivatives. *Arch. Pharm.* (Weinheim) **2019**, 352:e1800330.

2. P. Vrijens, S. Noppen, T. Boogaerts, E. Vanstreels, R. Ronca, P. Chiodelli, M. Laporte, E. Vanderlinden, S. Liekens, A. Stevaert, L. Naesens, Influenza virus entry via the GM3 ganglioside-mediated platelet-derived growth factor receptor beta signalling pathway. *J. Gen. Virol.* **2019**, *100*, 583-601.

^{1}H-DOSY spectra of compound 17

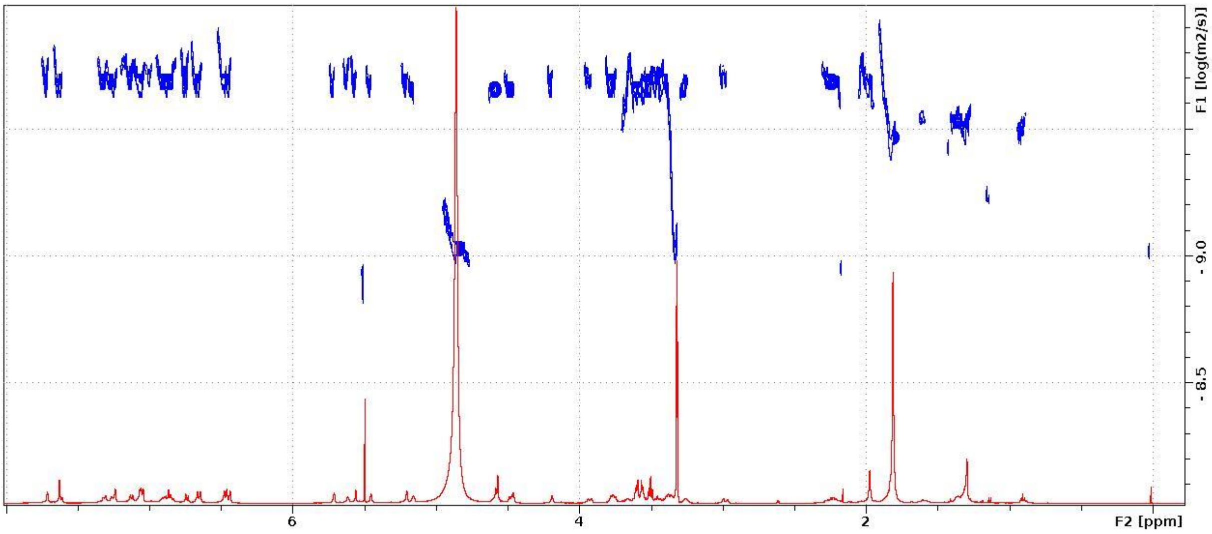

Figure S1. Detection of possible oligomerisation of **17** by DOSY (Diffusion Ordered Spectroscopy) NMR experiments with TMS as internal reference. On this scale 0.1 unit accords to double mass ratio. The vertical scale represent diffusion constant on a log10 scale. This experiment yielded MW between 7- 8 kDa

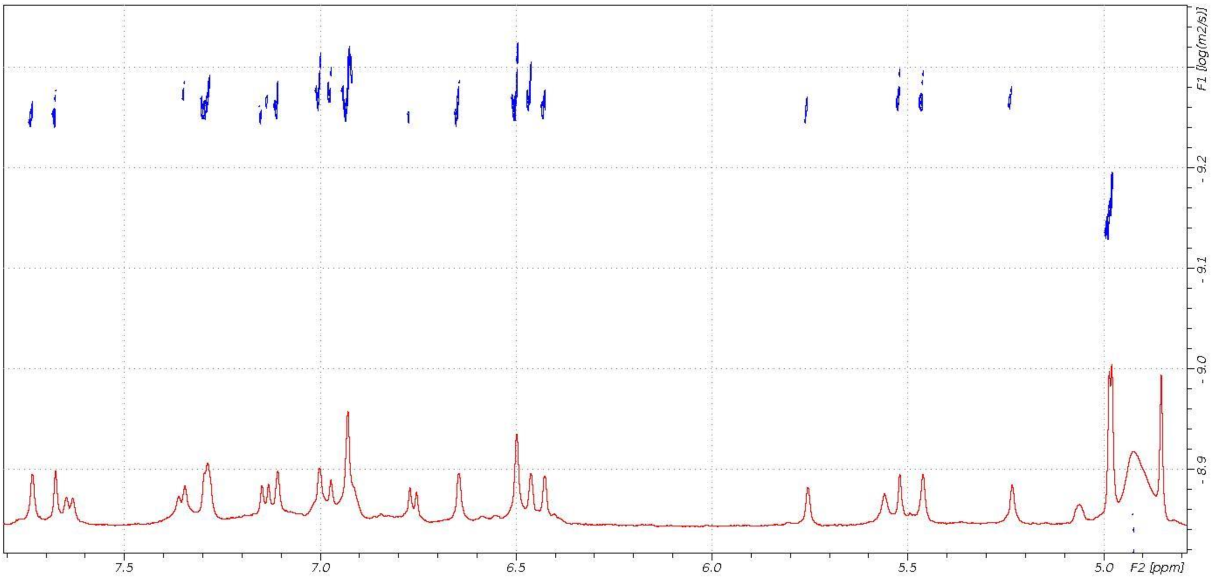

Figure S2. DOSY NMR experiment of **17** in MeOD with β-CD as internal reference, the vertical scale is a log10 based diffusion constant. On this scale 0.1 unit is a mass factor of two. β-CD (anomeric doublet in 1D spectrum, bottom at 5 ppm) appears at - 9.15 while the **17** front at - 9.26. This results in mass 2400 (instead of nominal 2030), according to rather a monomeric mass

NMR Spectra of the compounds

^{1}H NMR and ^{13}C NMR spectra of compound **2a**

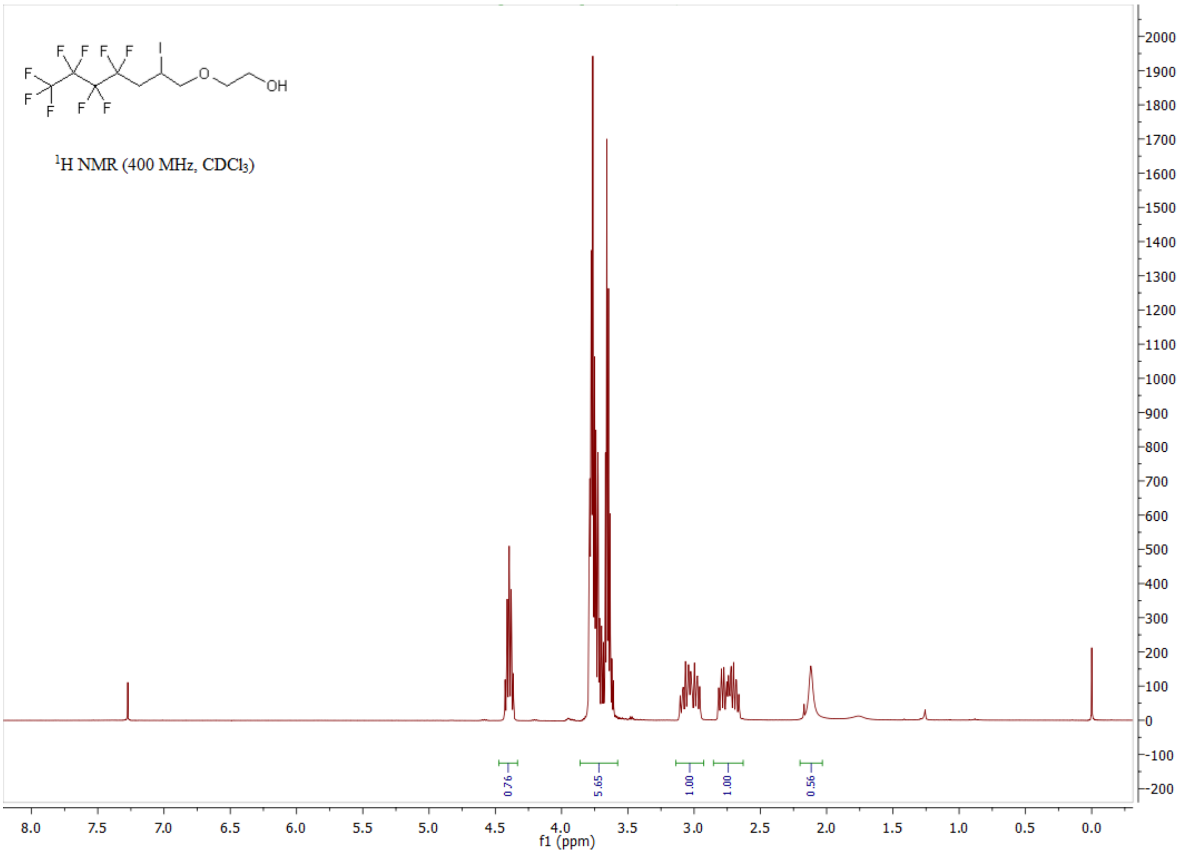

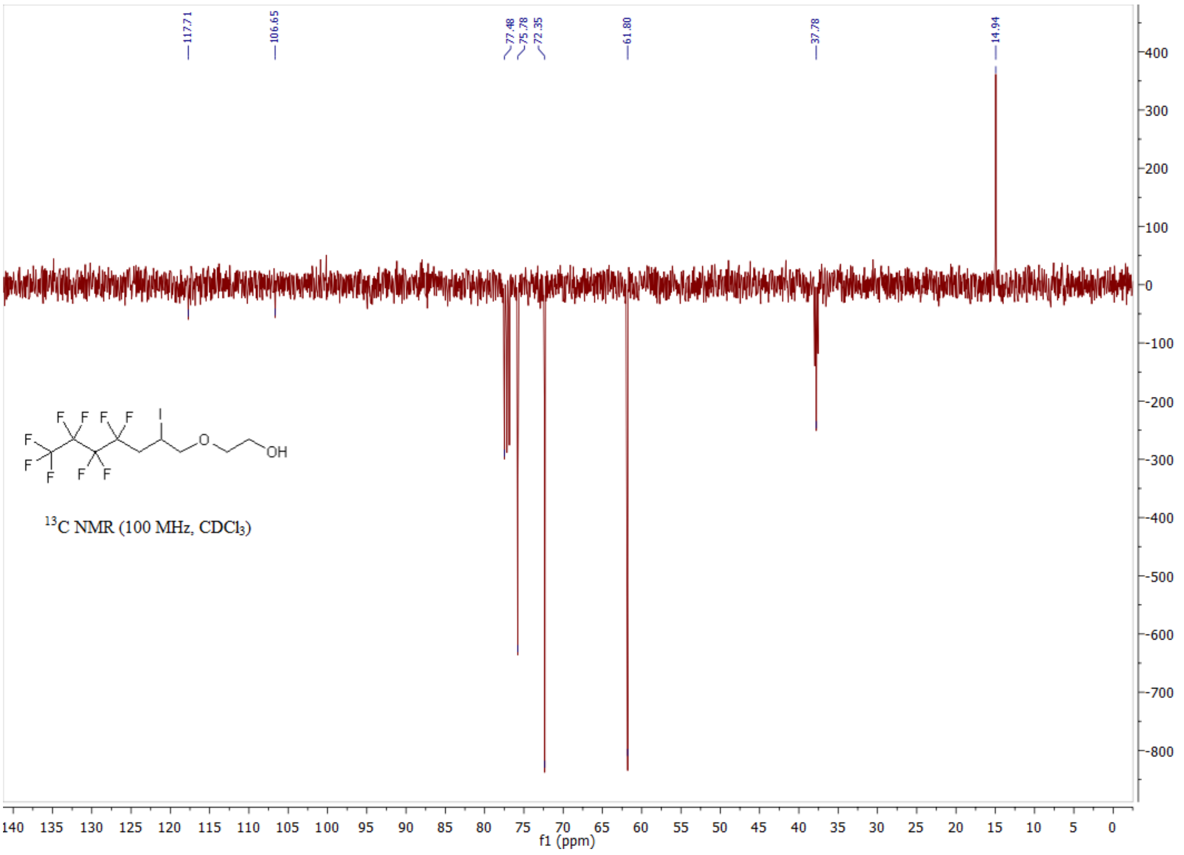

^{1}H NMR and ^{13}C NMR spectra of compound **2b**

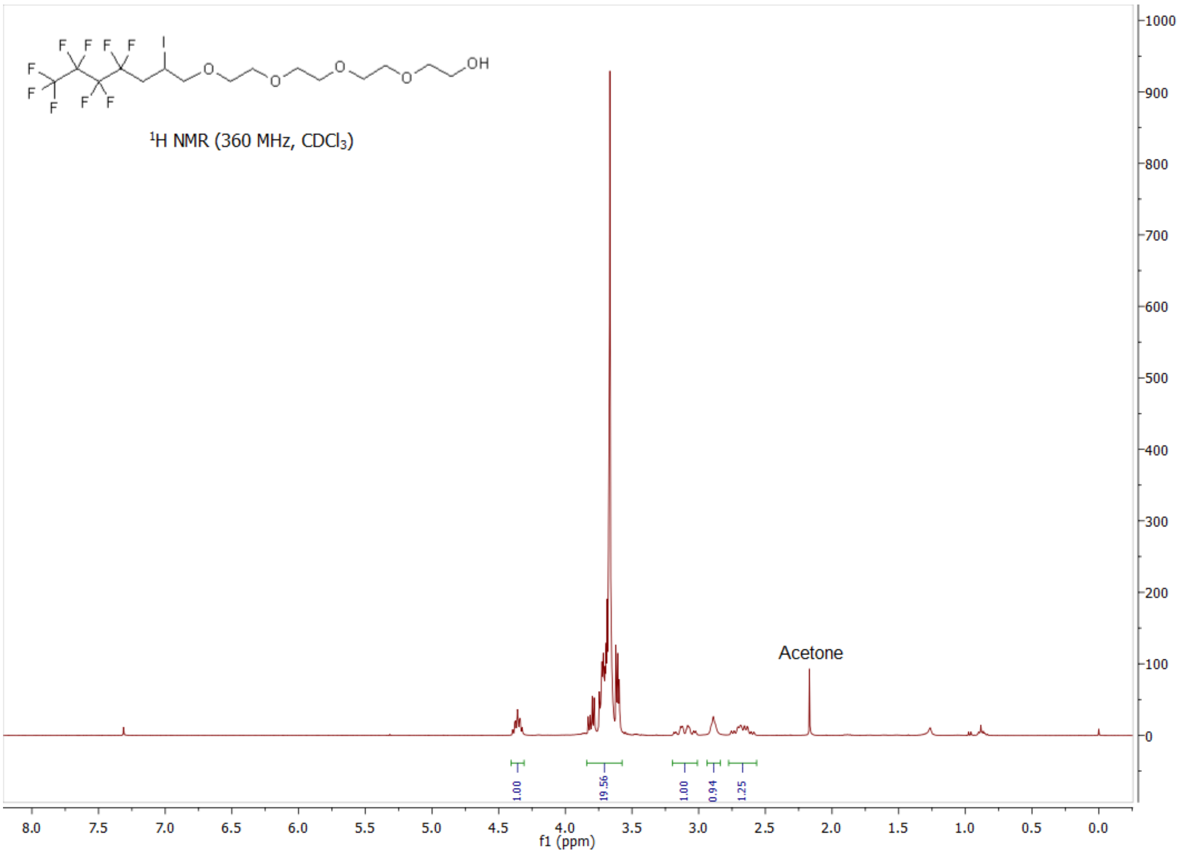

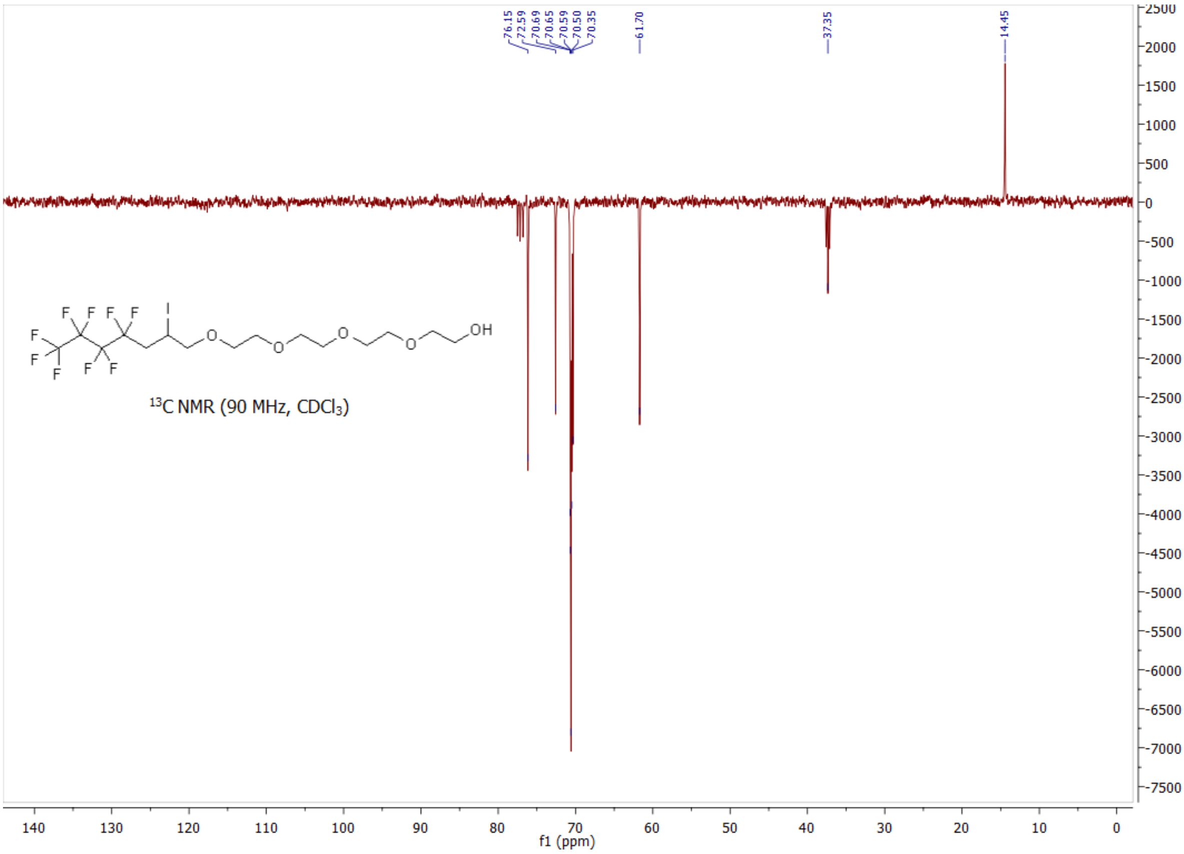

^{1}H NMR and ^{13}C NMR spectra of compound **3a**

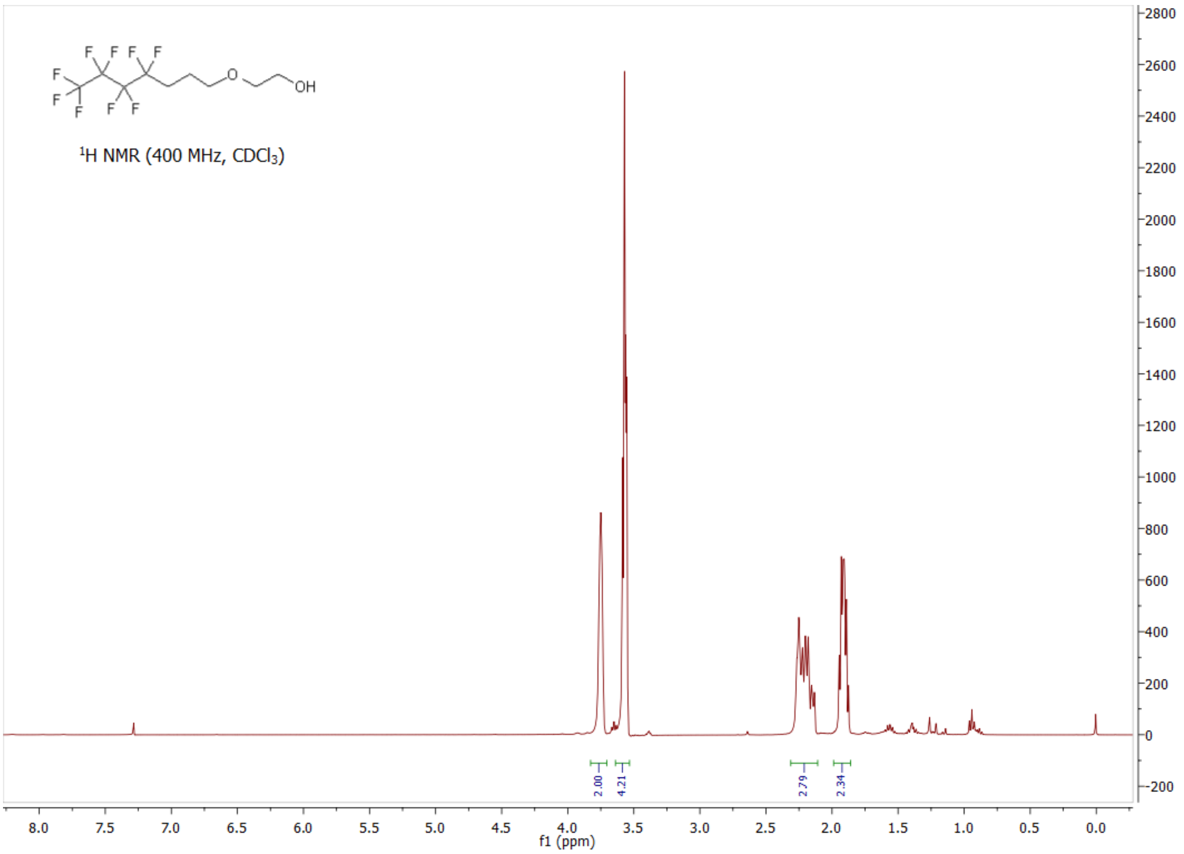

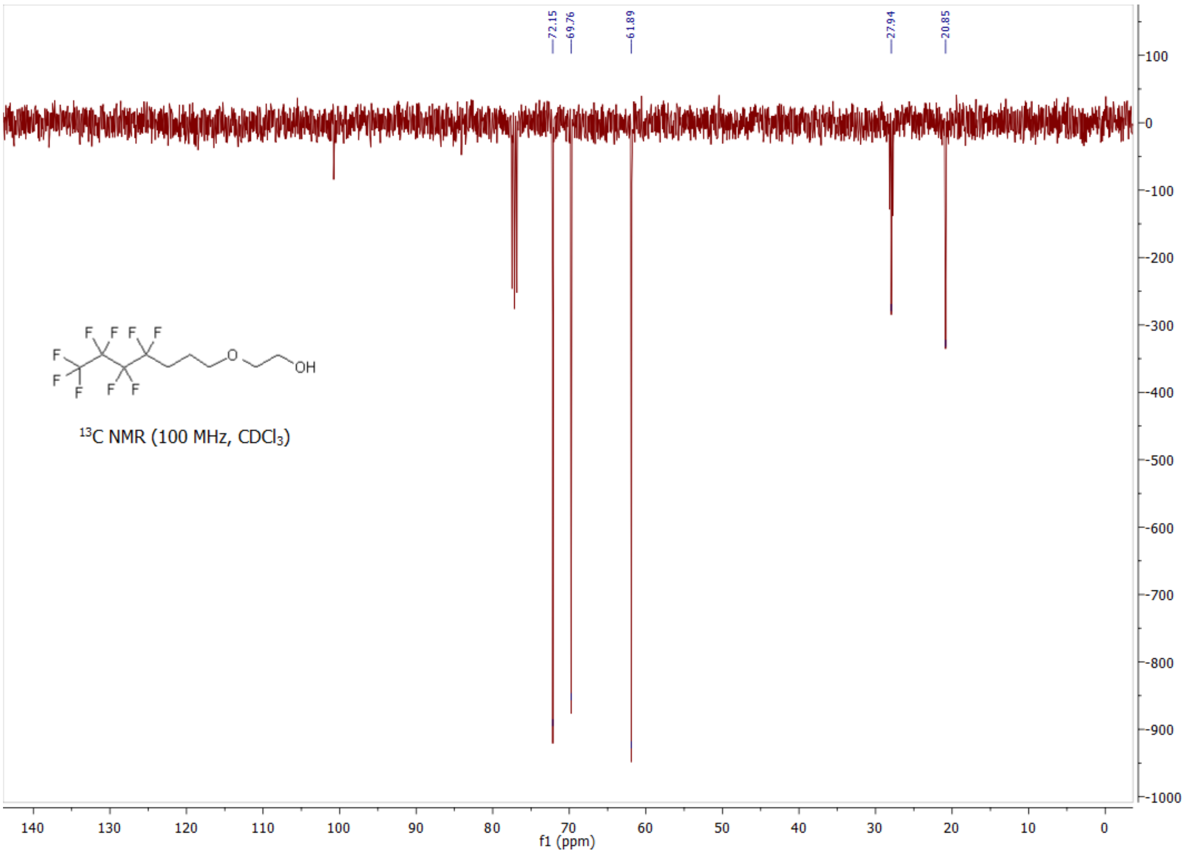

^{1}H NMR and ^{13}C NMR spectra of compound **3b**

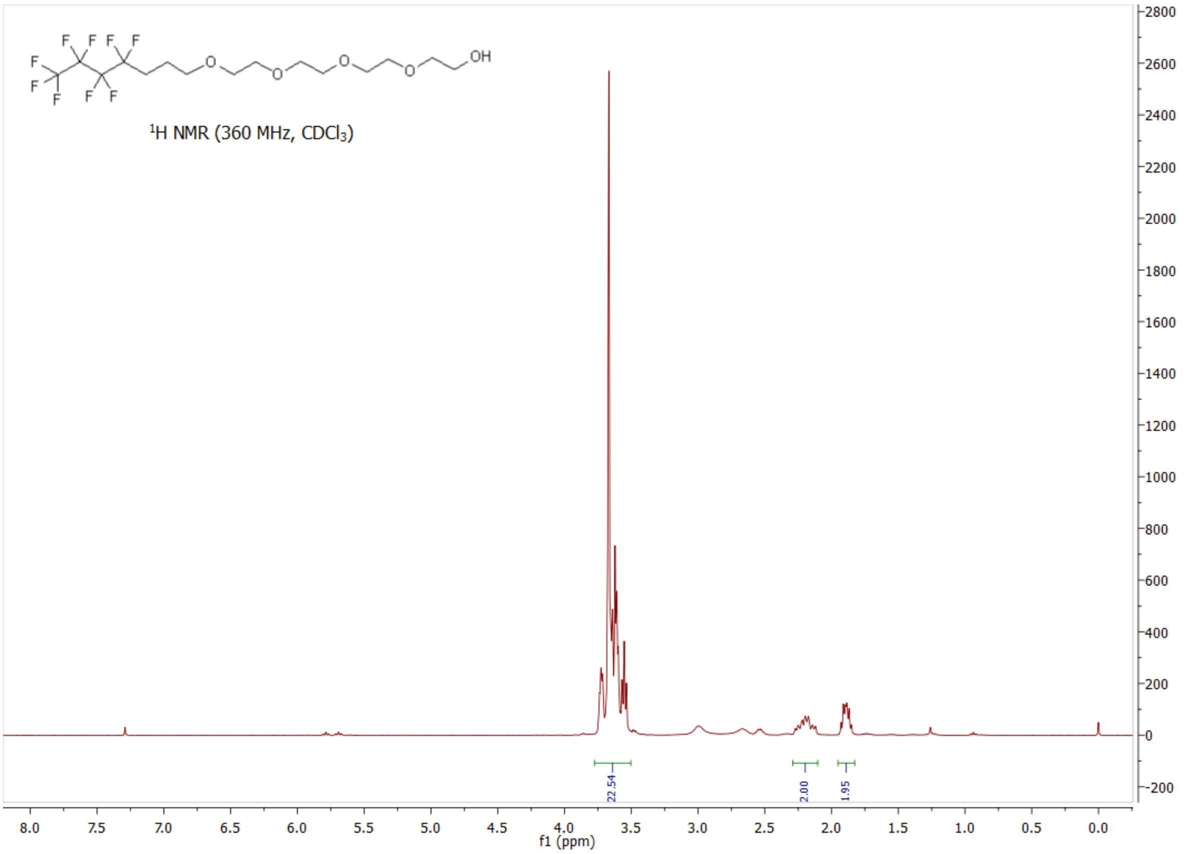

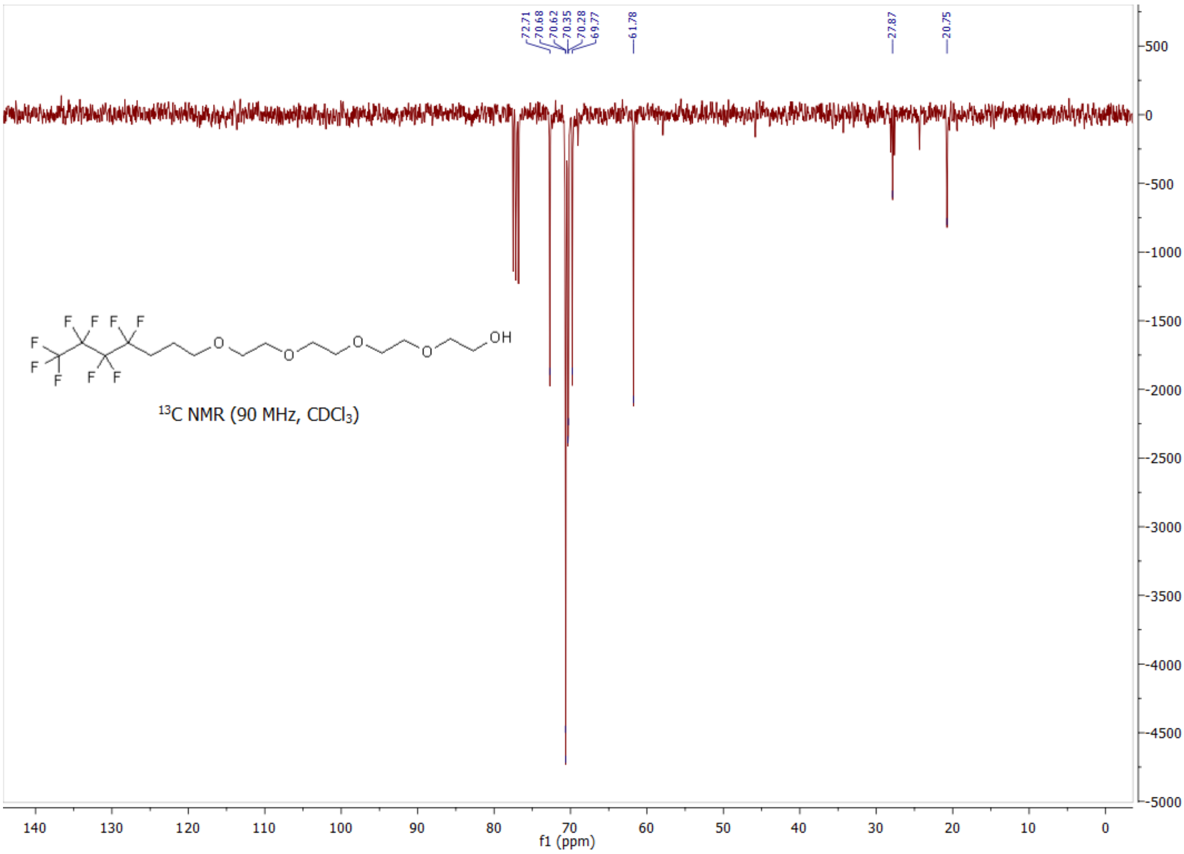

^{1}H NMR and ^{13}C NMR spectra of compound **4a**

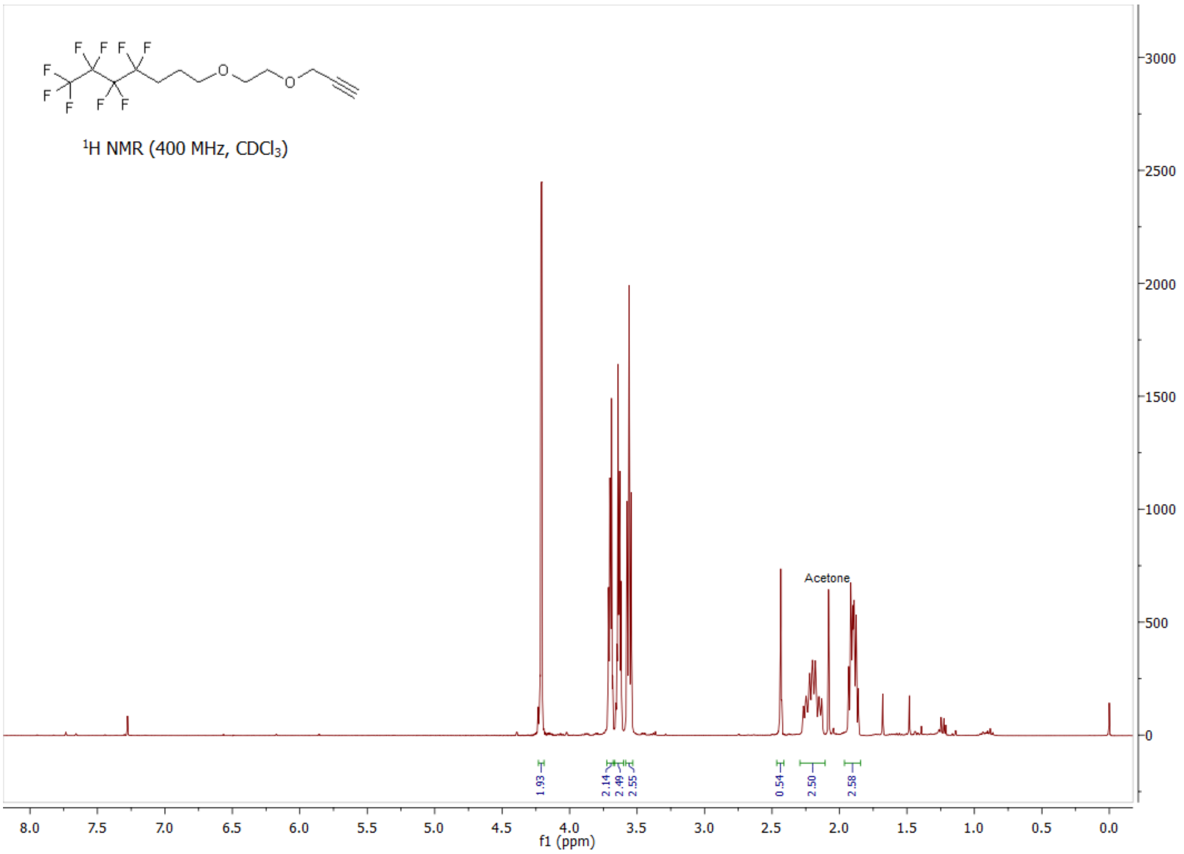

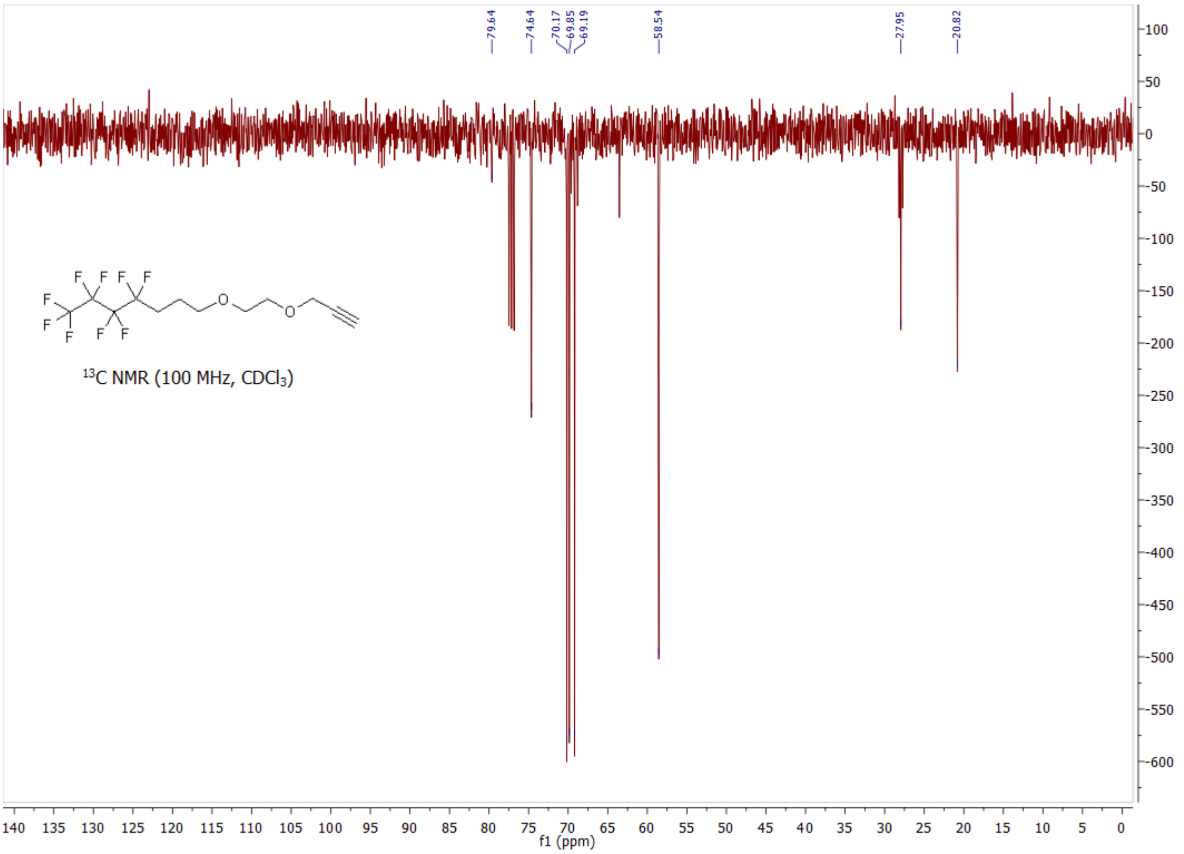

^{1}H NMR and ^{13}C NMR spectra of compound **4b**

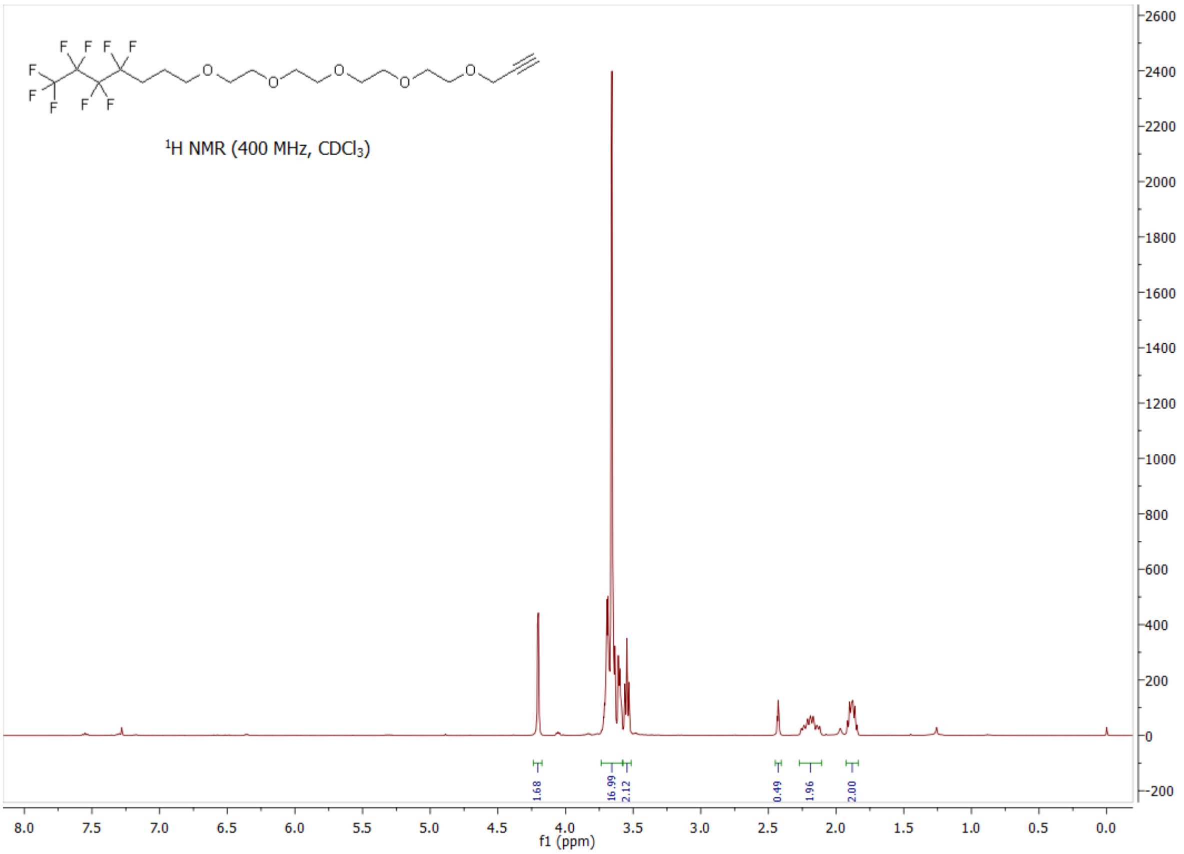

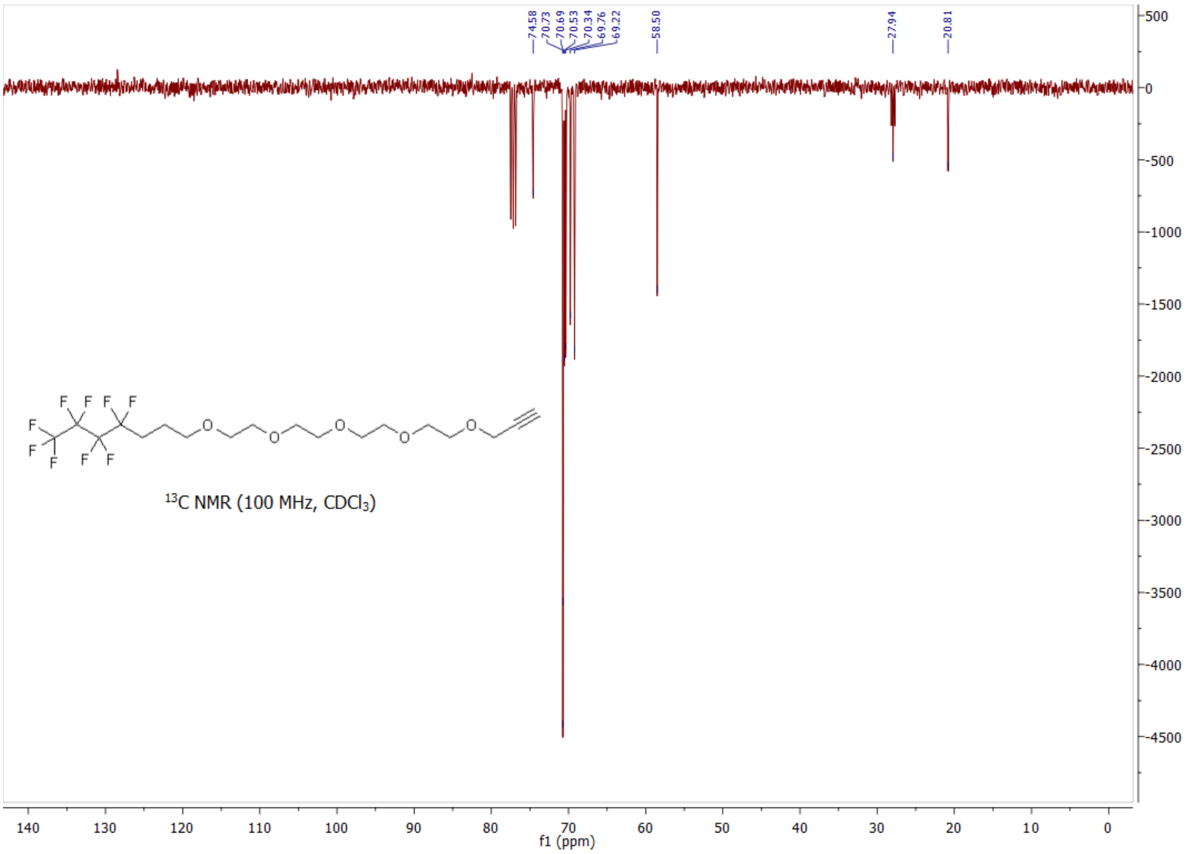

^{1}H NMR and ^{13}C NMR spectra of compound **5a**

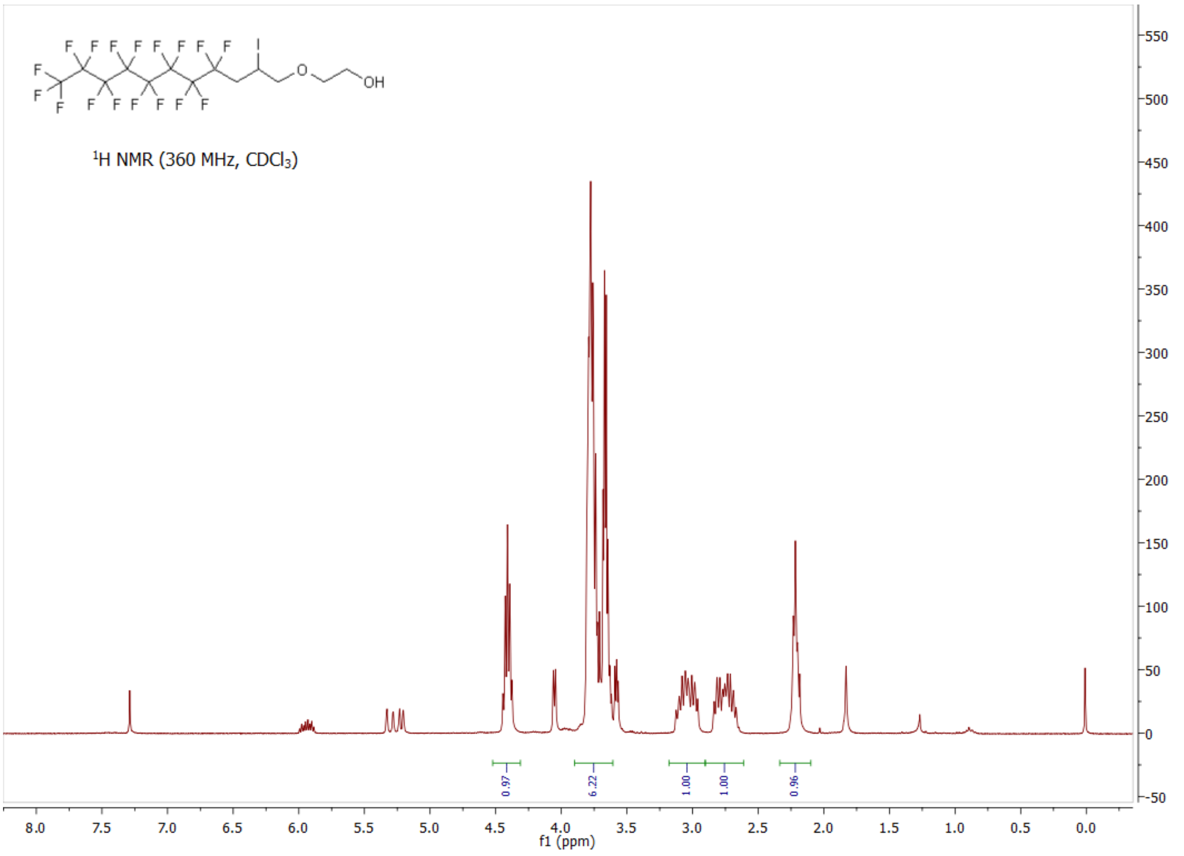

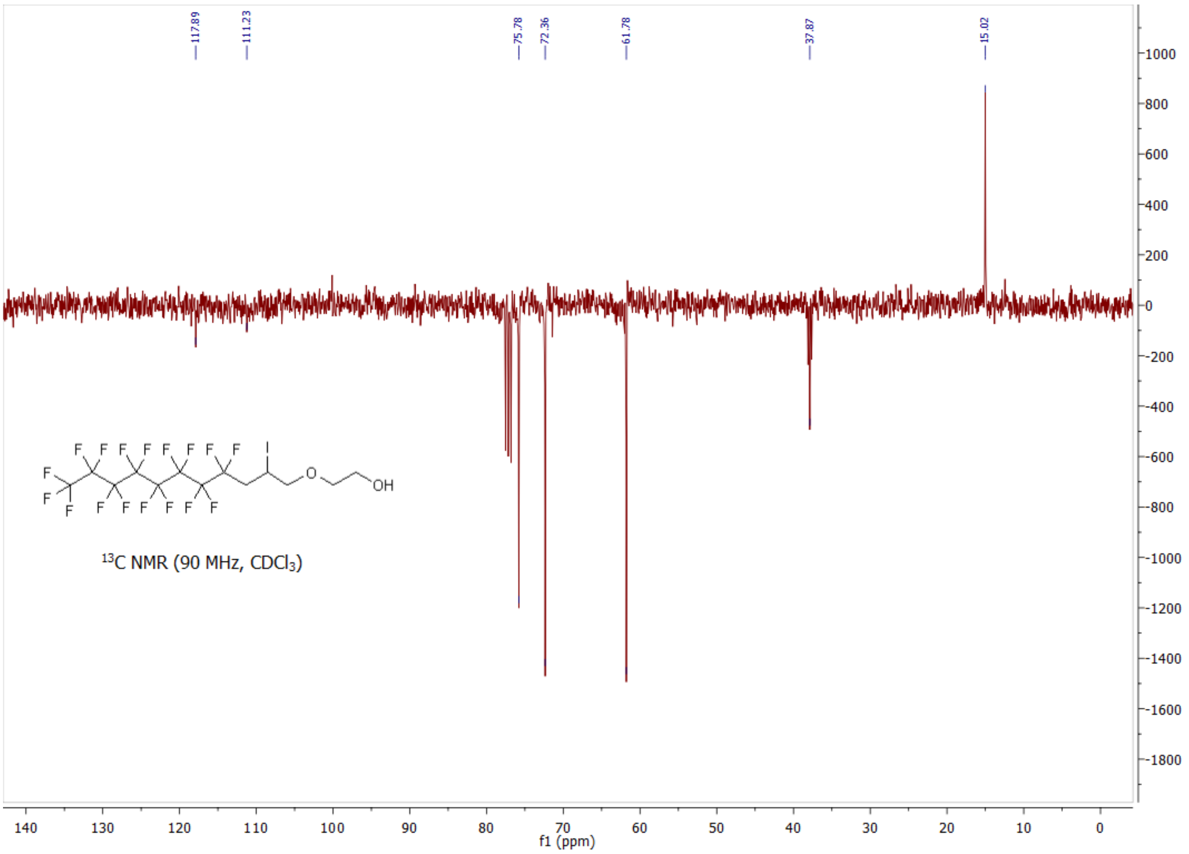

^{1}H NMR and ^{13}C NMR spectra of compound **5b**

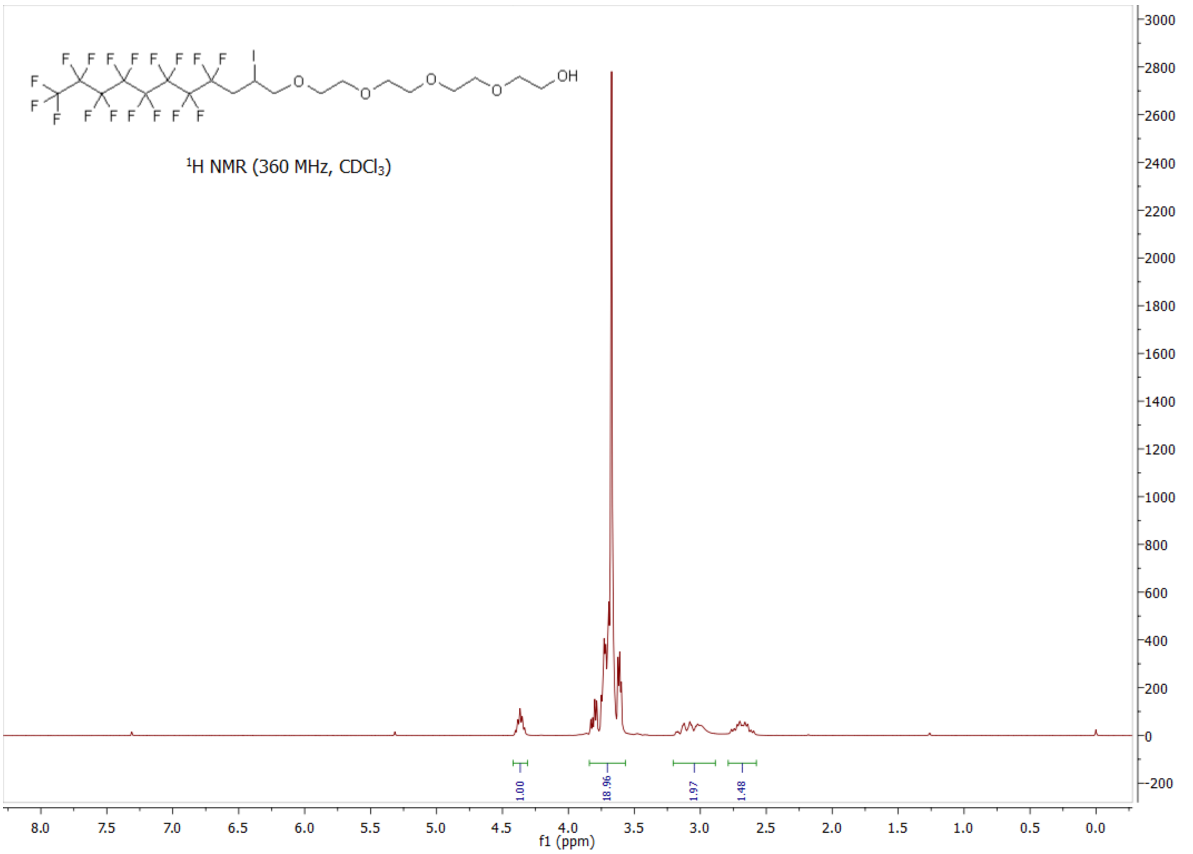

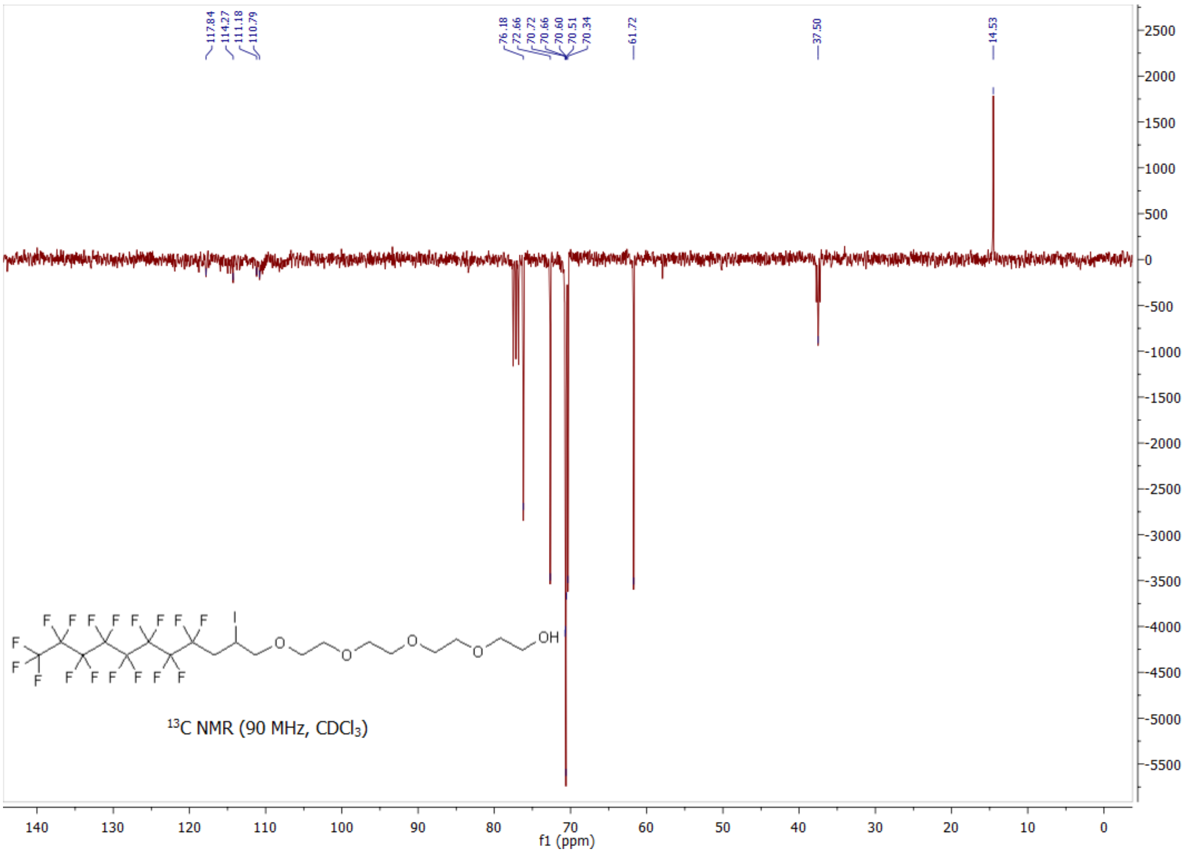

^{1}H NMR and ^{13}C NMR spectra of compound **6a**

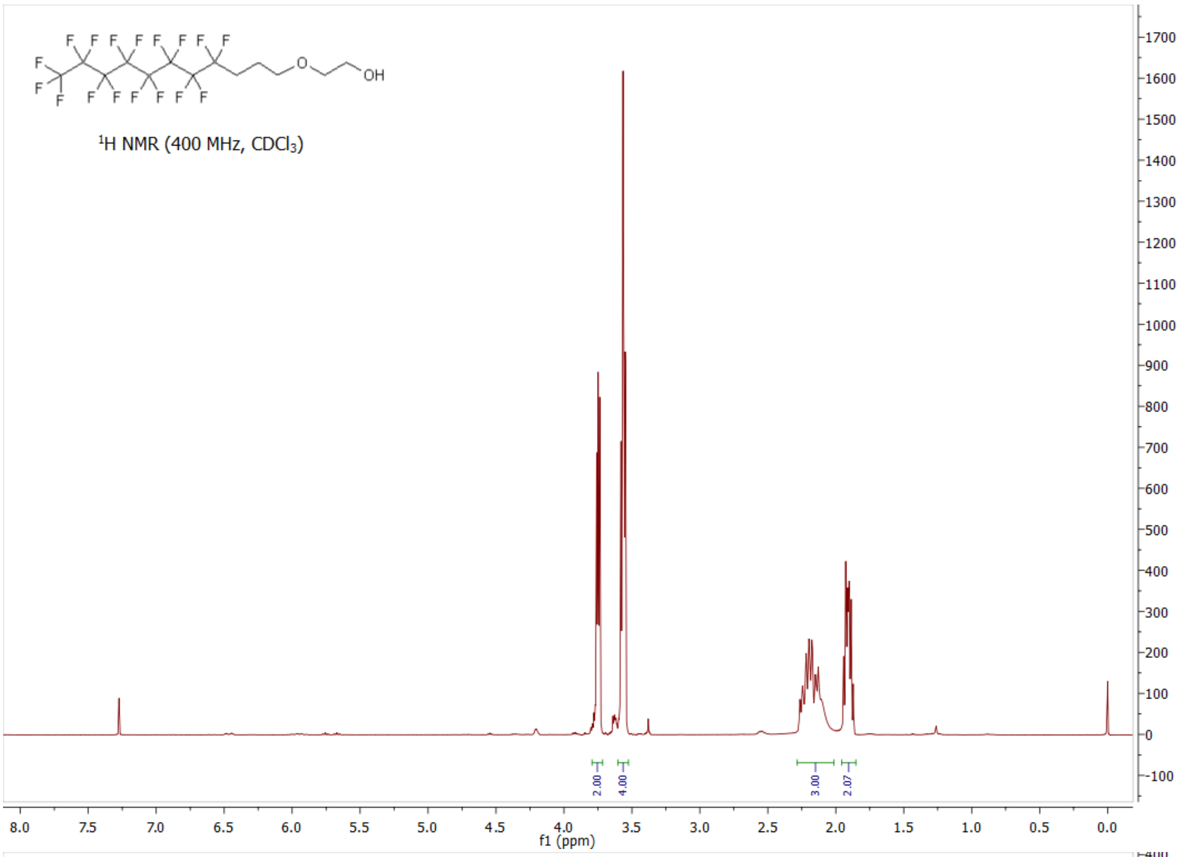

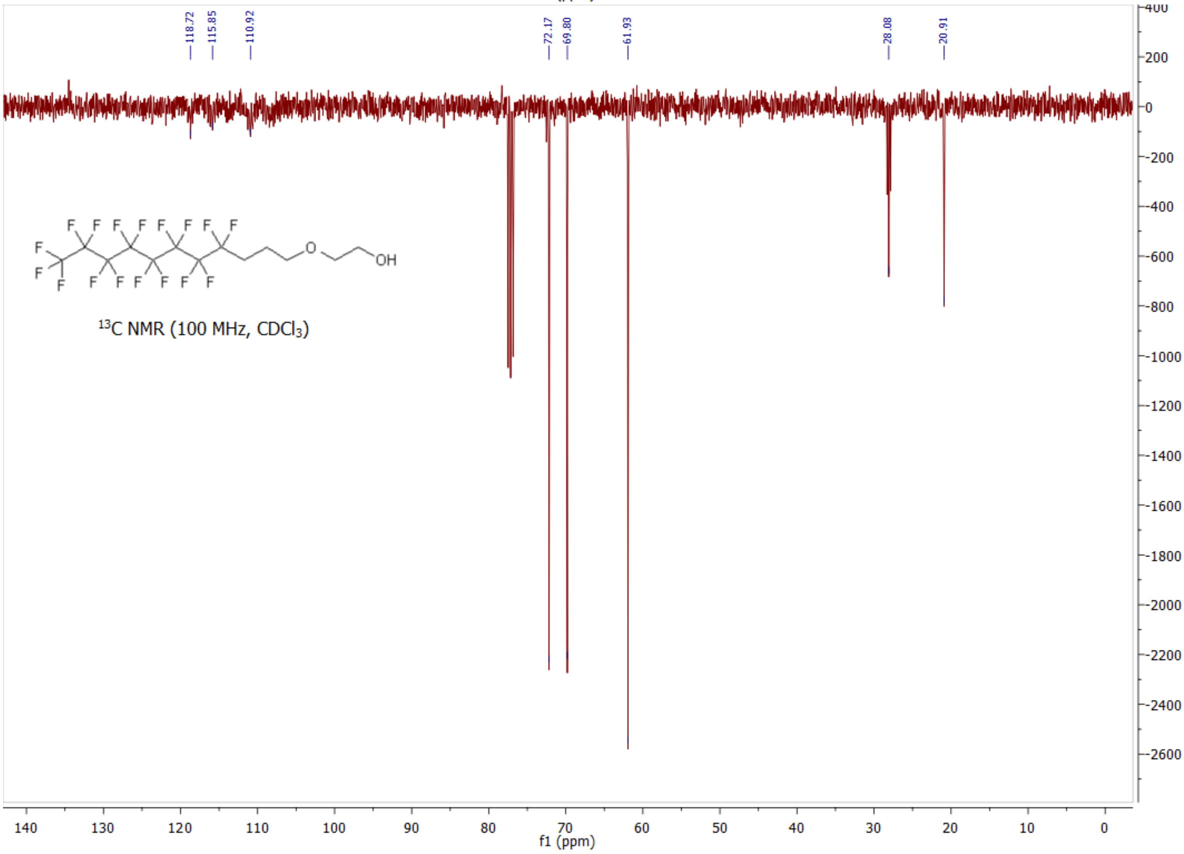

^{1}H NMR and ^{13}C NMR spectra of compound **6b**

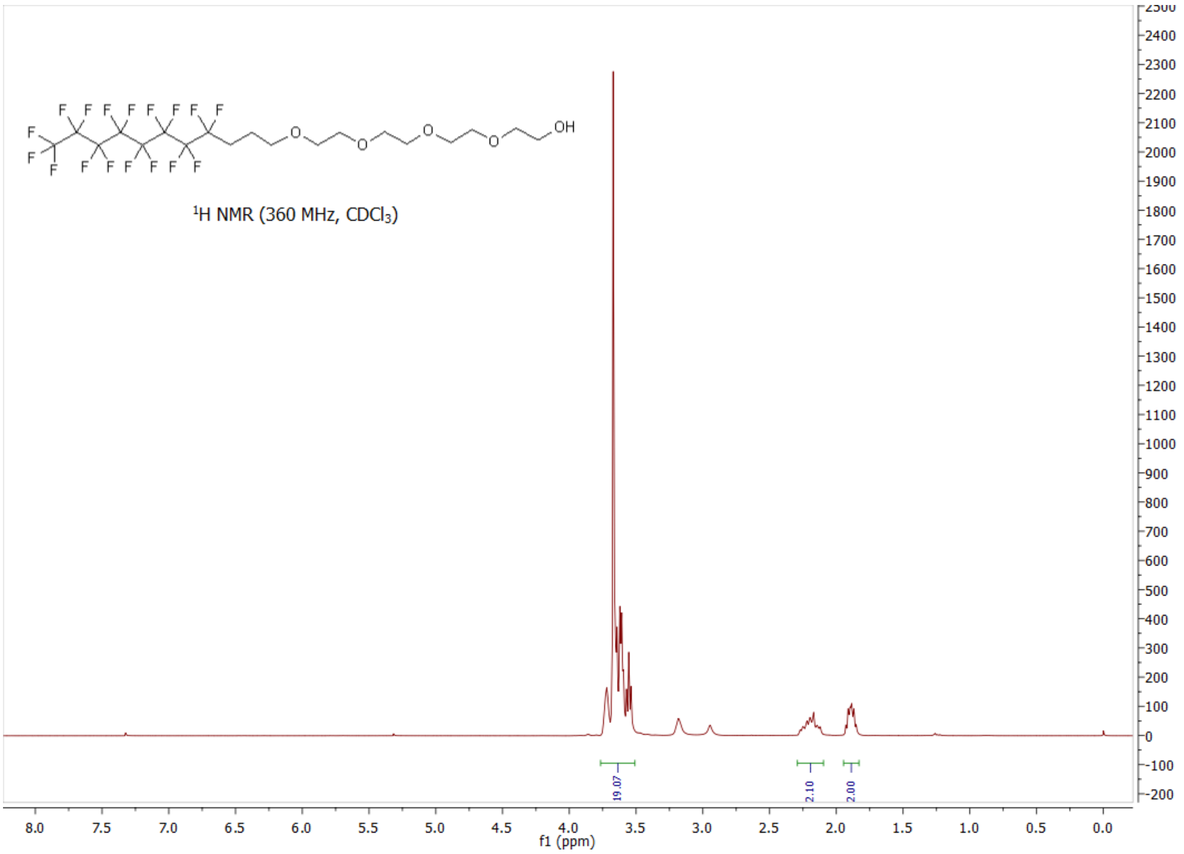

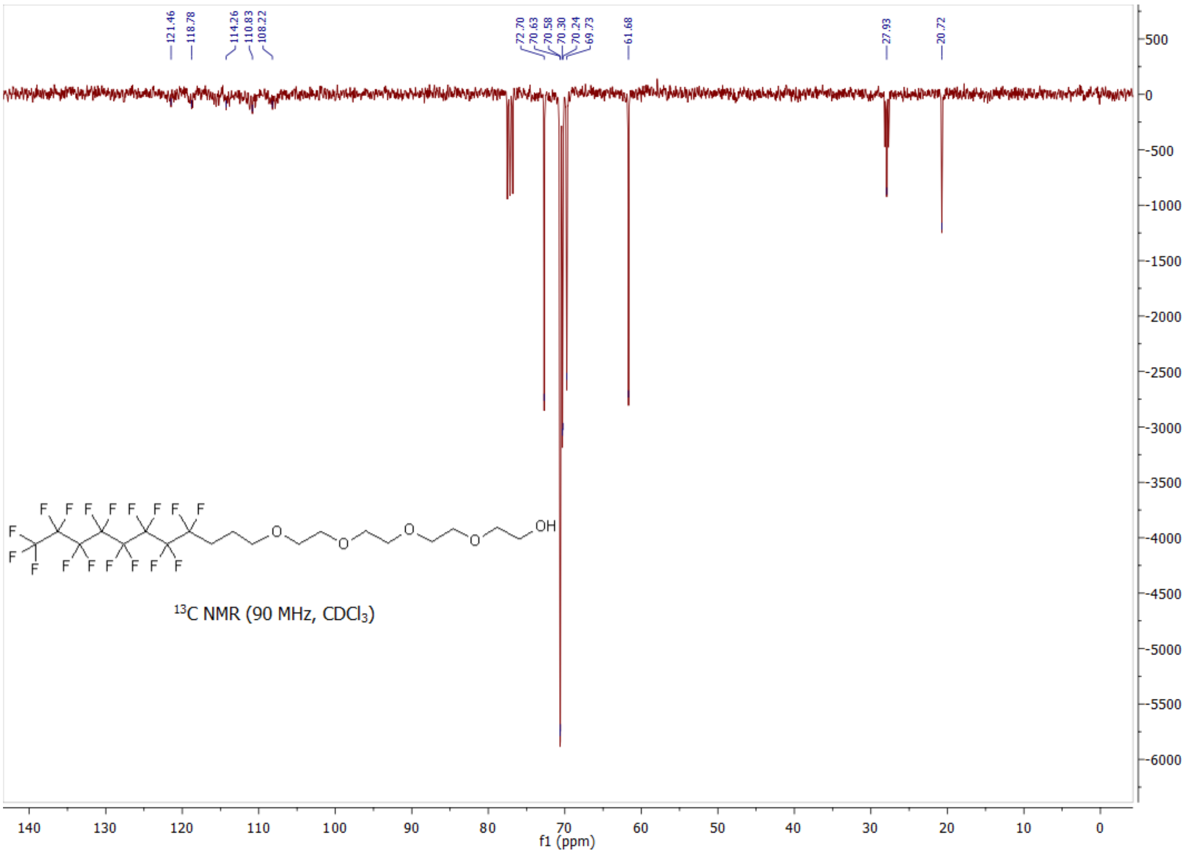

^{1}H NMR and ^{13}C NMR spectra of compound **7a**

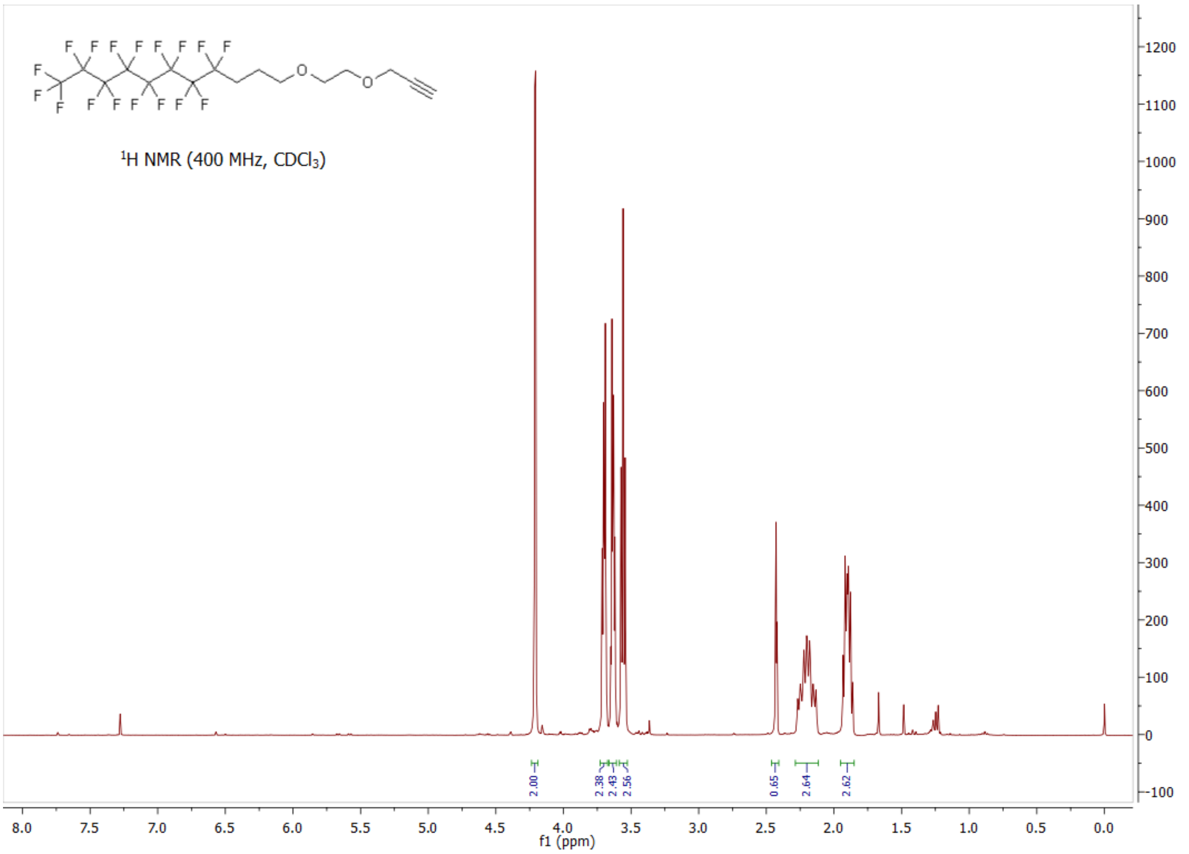

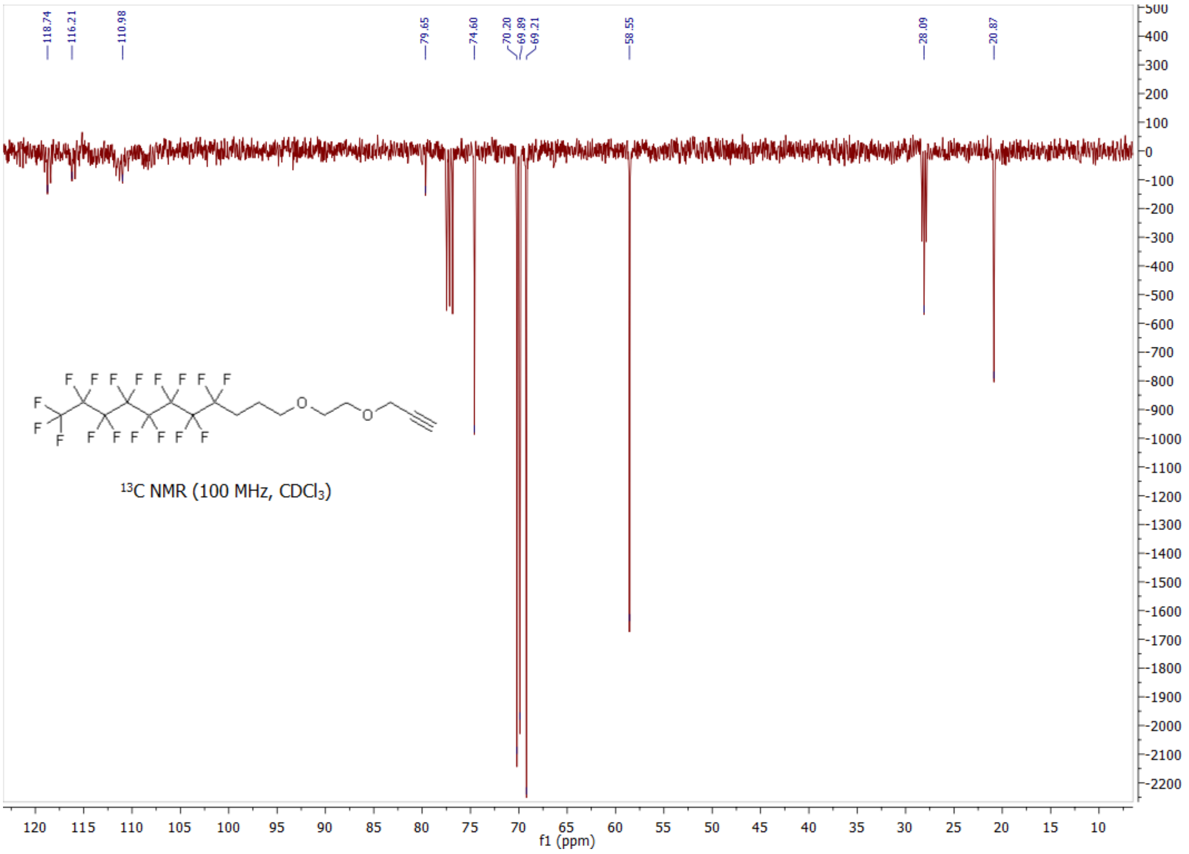

^{1}H NMR and ^{13}C NMR spectra of compound **10**

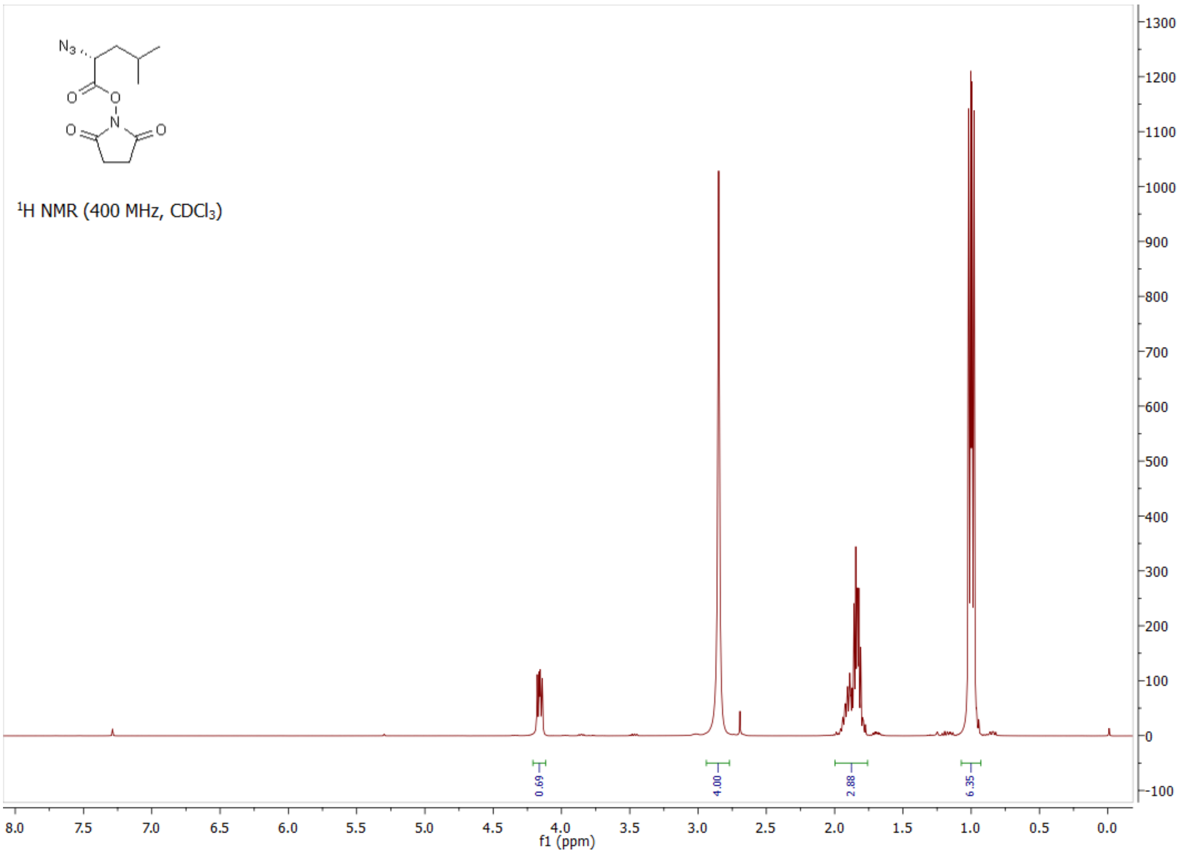

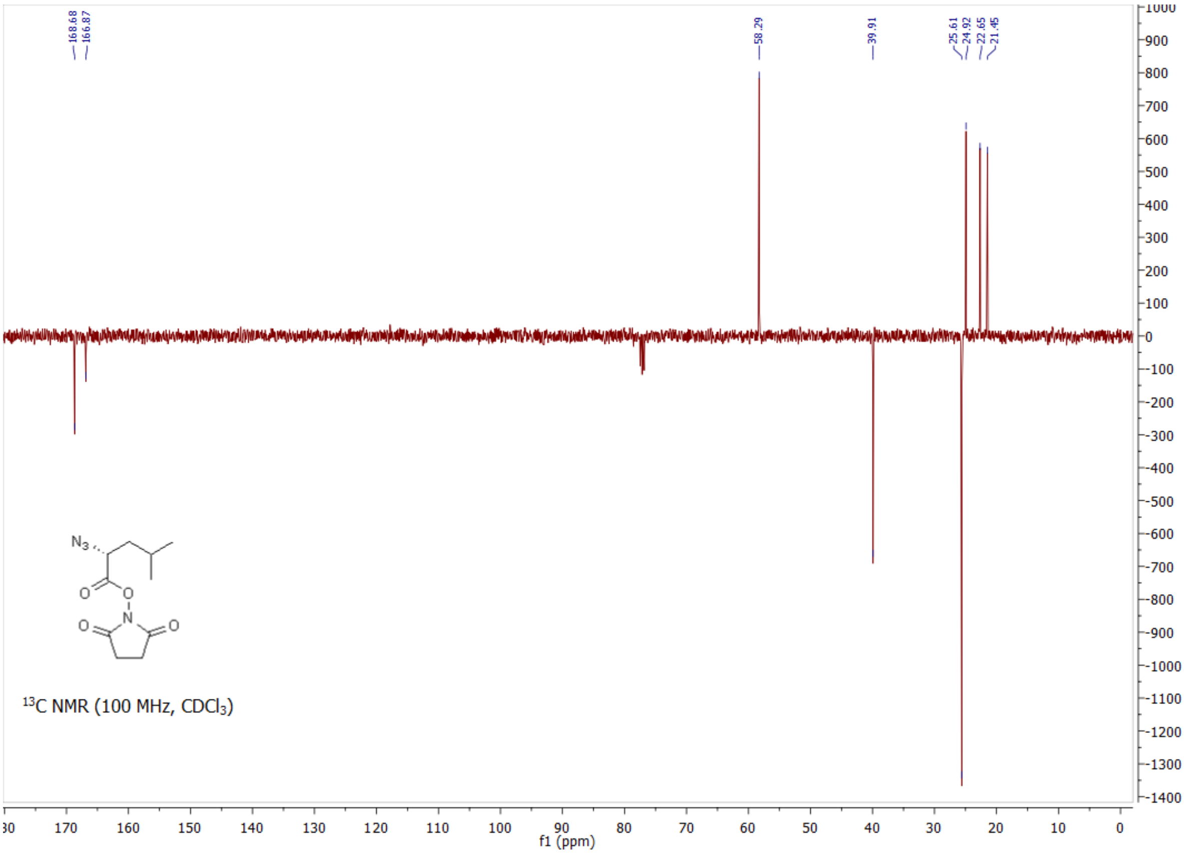

JMOD, HSQC and HMBC spectra of compound **11**, (125, 500 MHz) DMSO

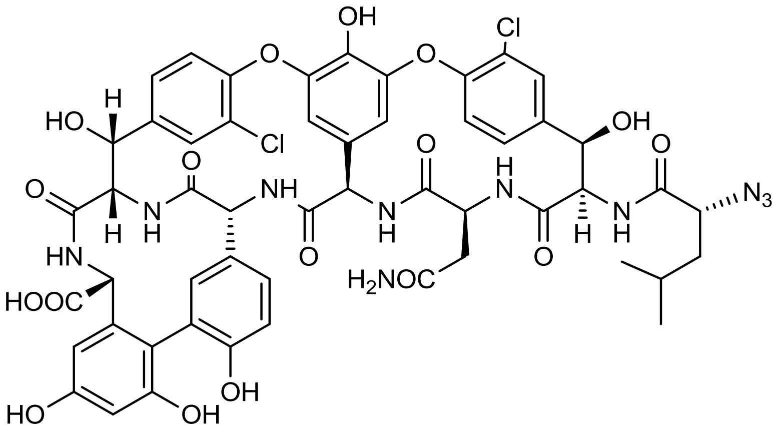

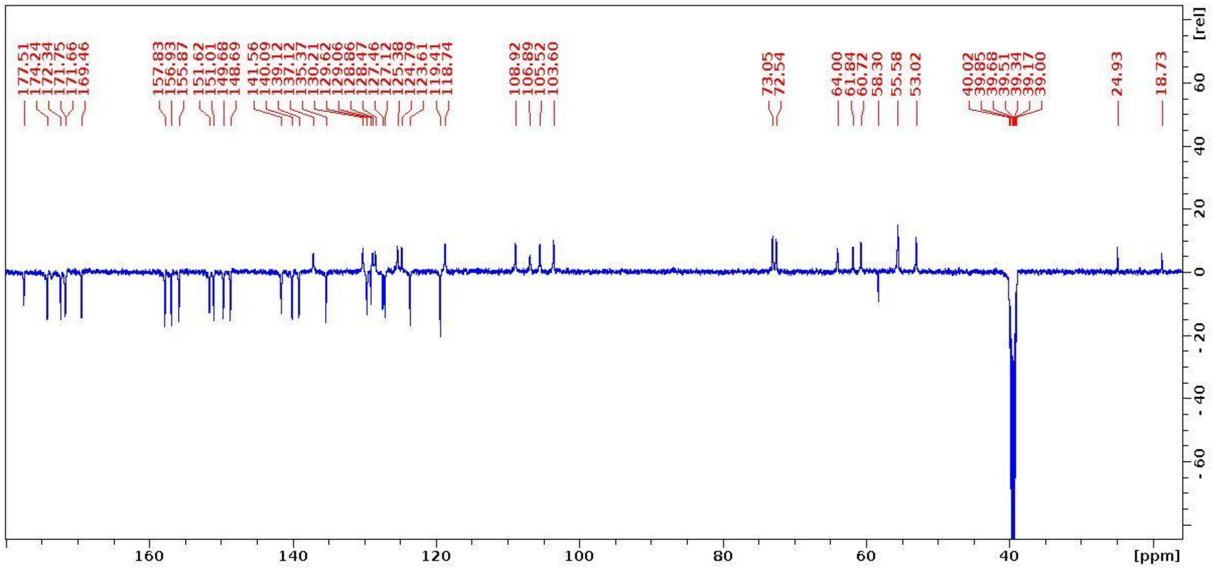

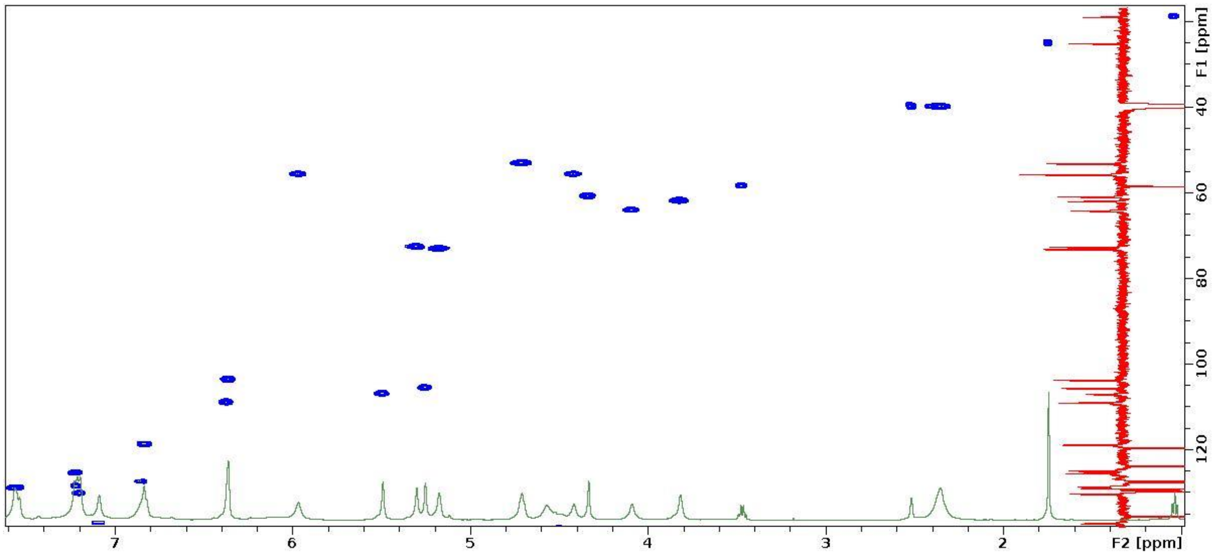

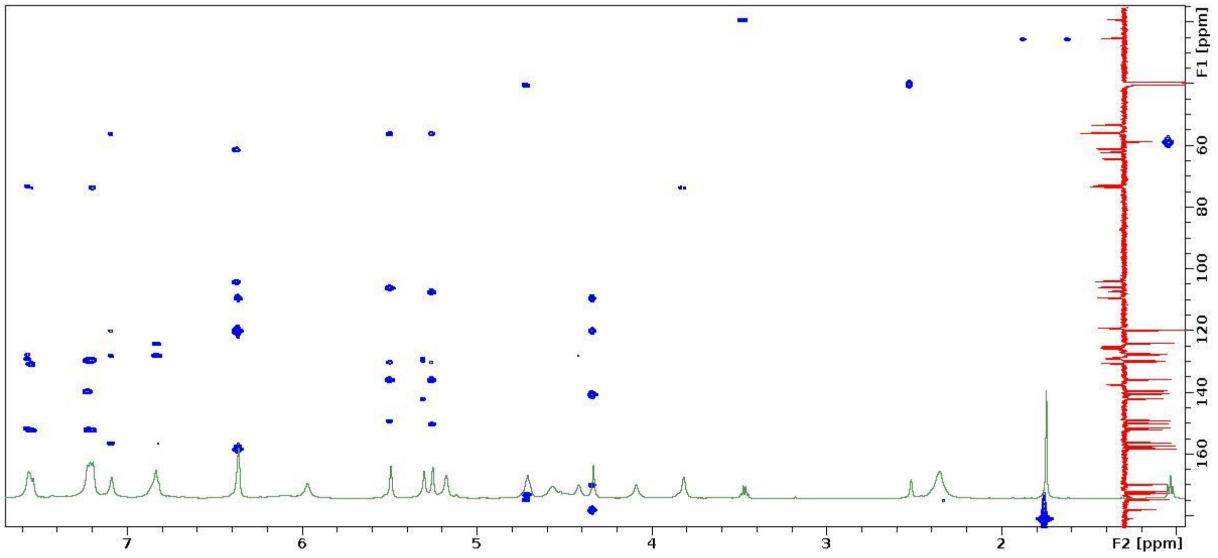

^{13}C, HSQC and HMBC spectra of compound **12**, (125, 500 MHz) DMSO

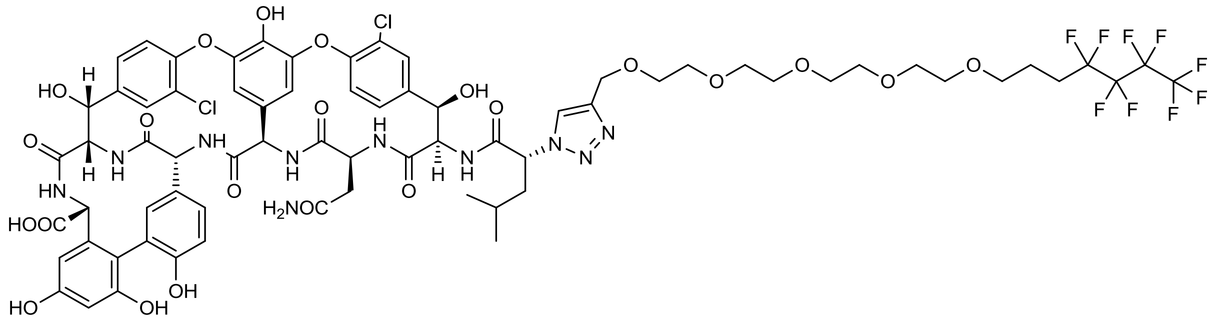

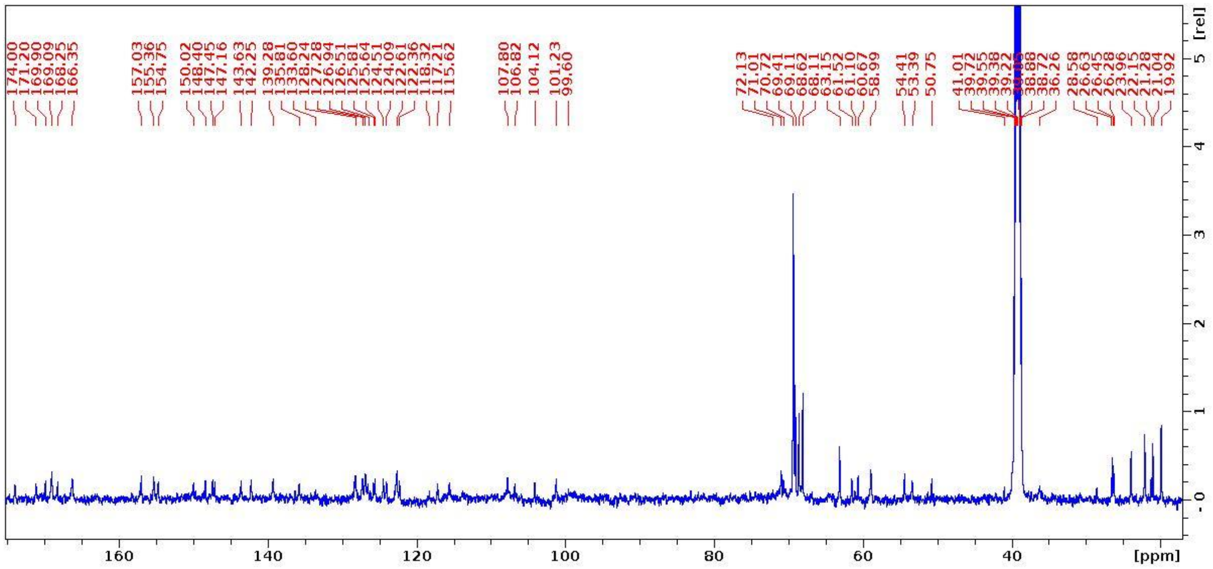

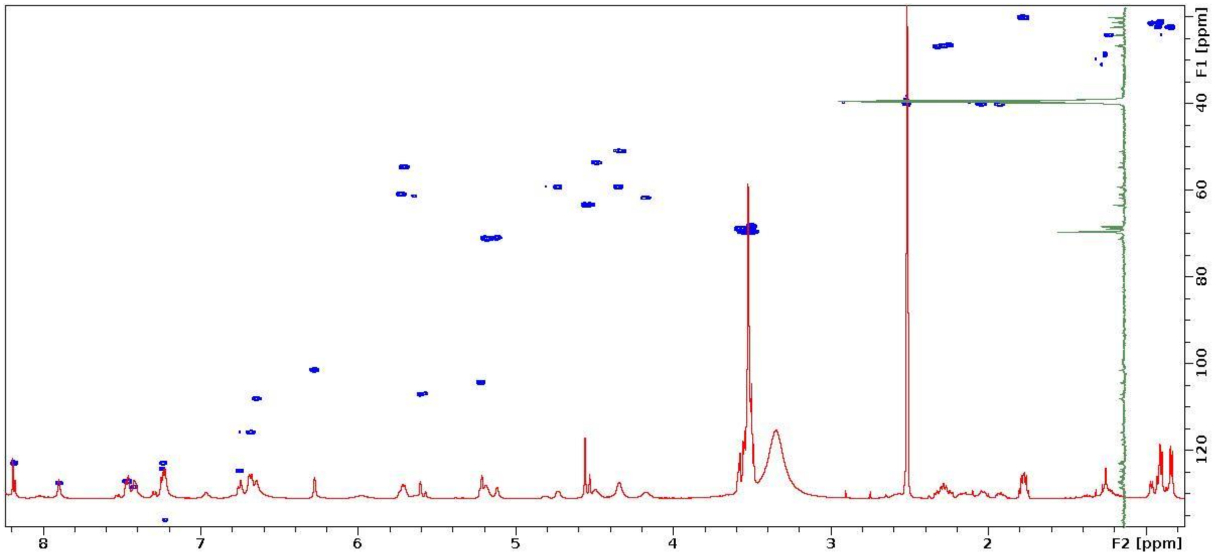
F1 [ppm]
F2 [ppm]

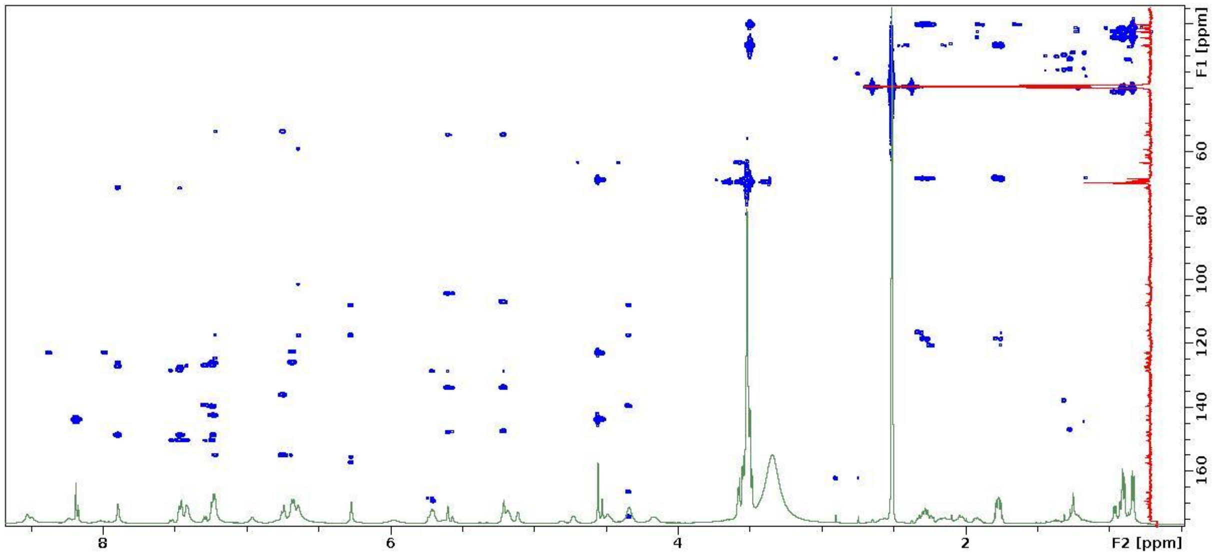
F1 [ppm]
F2 [ppm]

^{19}F, JMOD and HSQC spectra of compound **13**, (470, 125, 500 MHz) DMSO

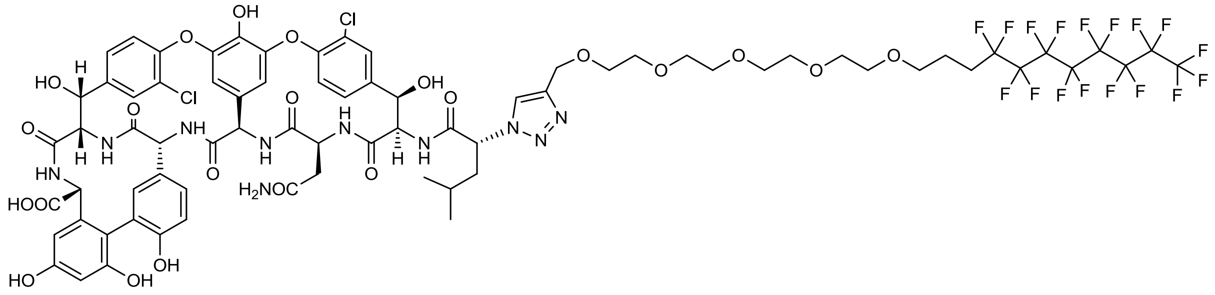

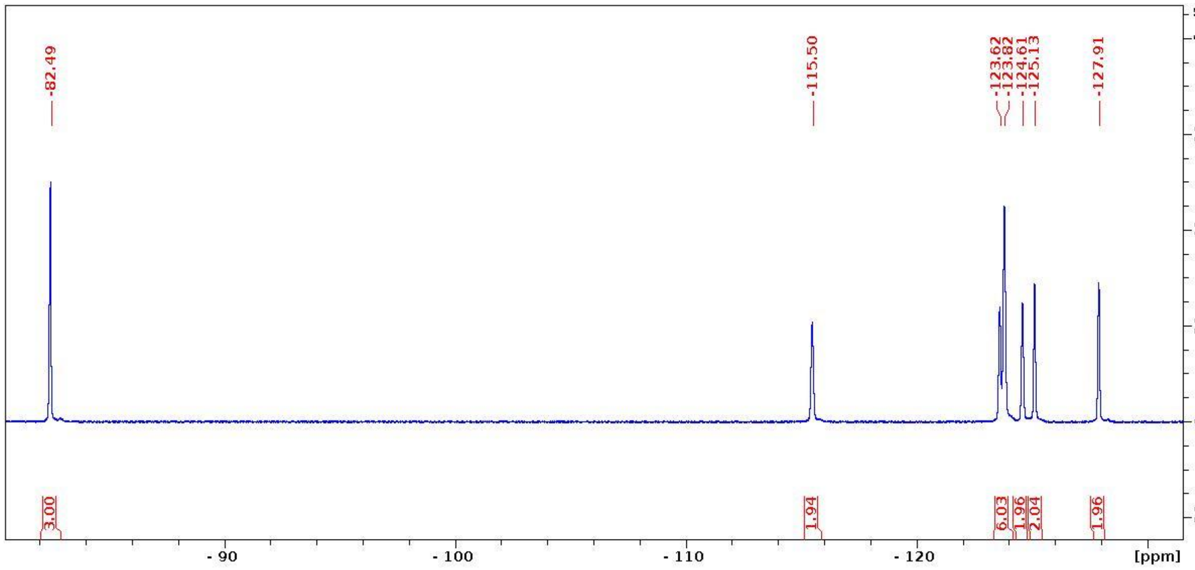

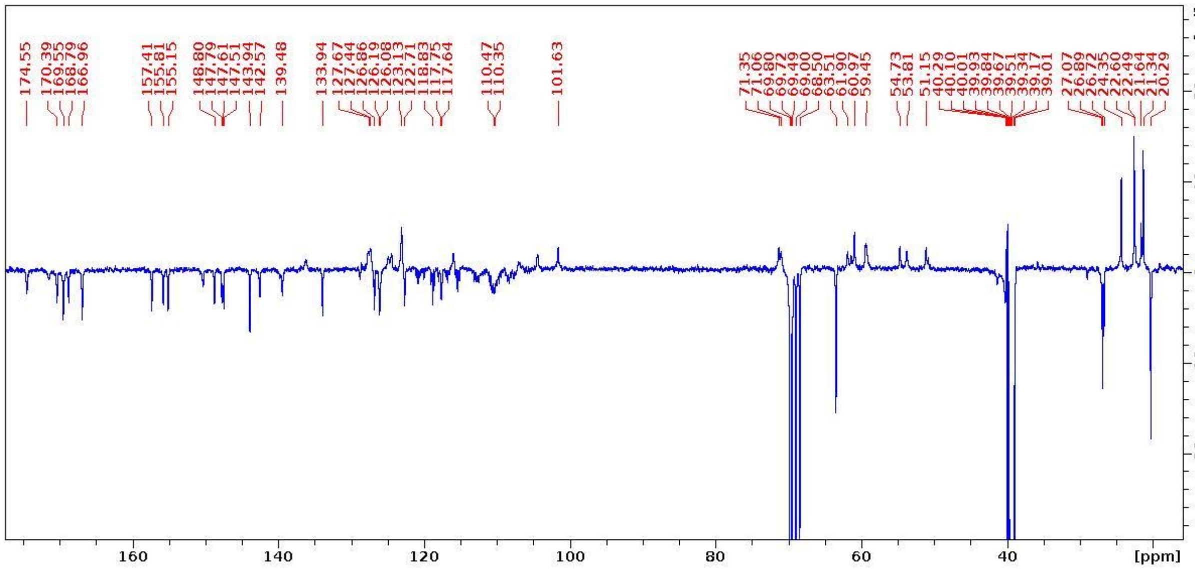

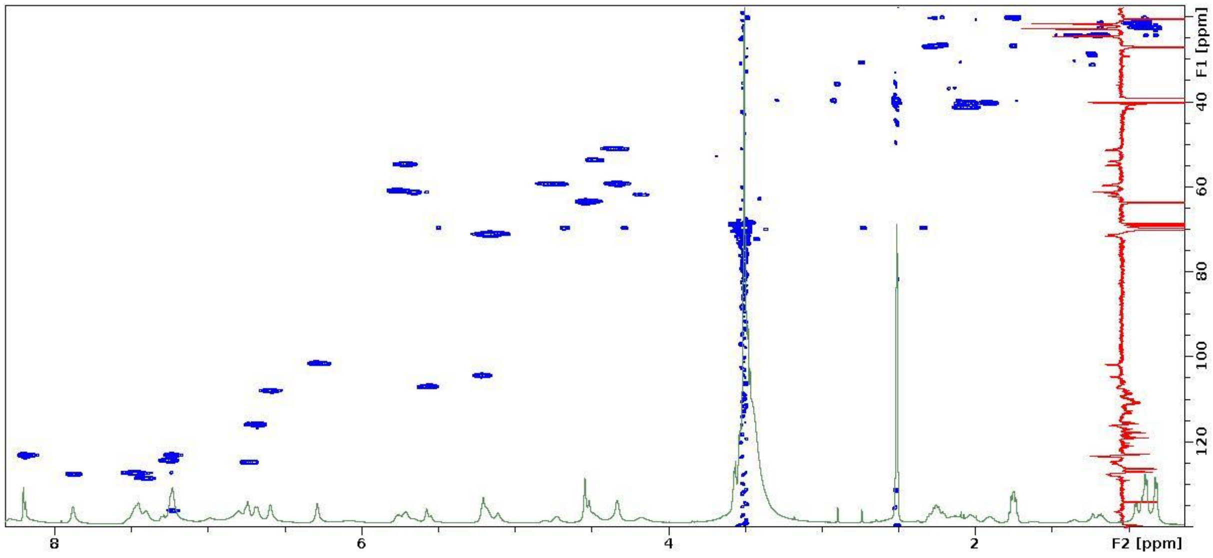

JMOD, HSQC and HMBC spectra of compound **15**, (125, 500 MHz) DMSO

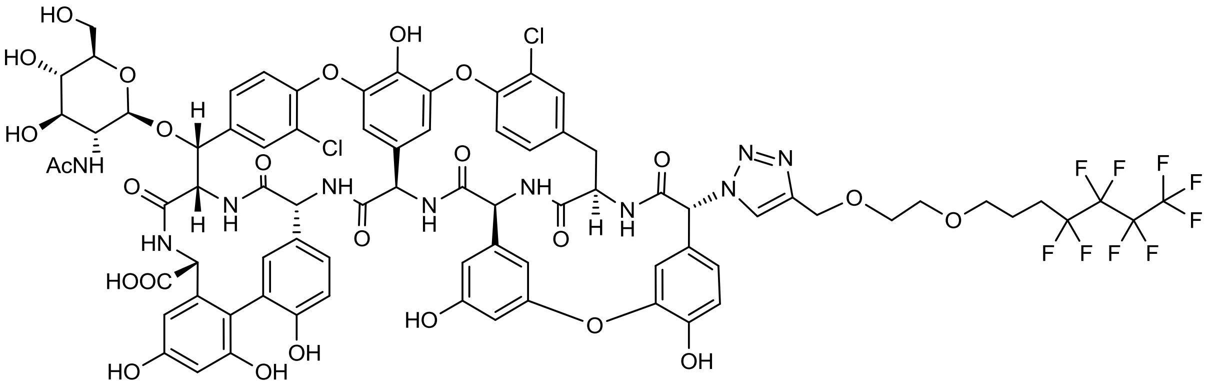

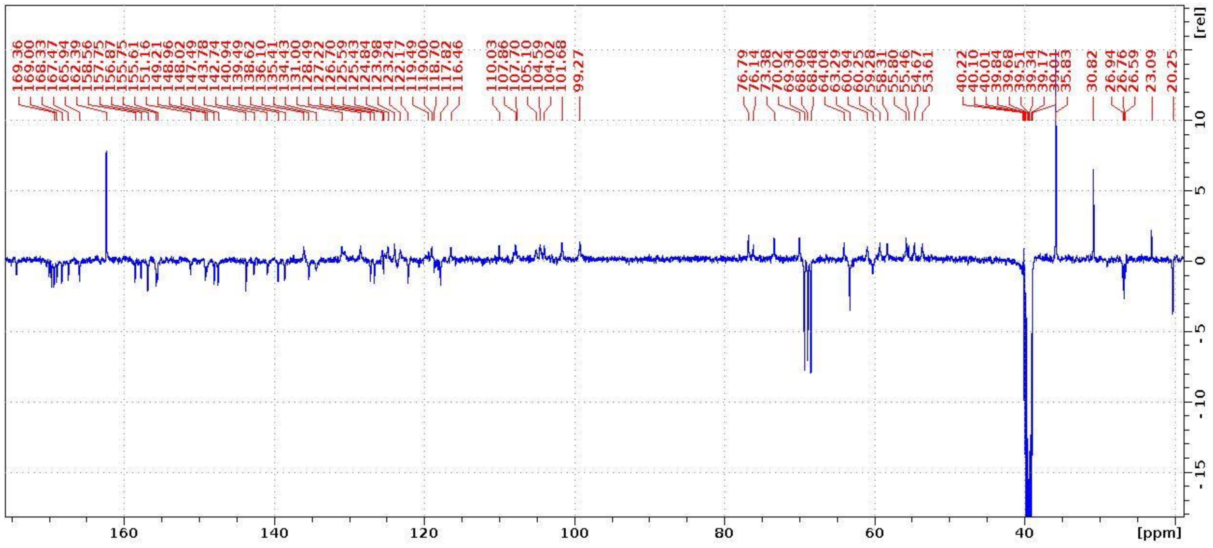

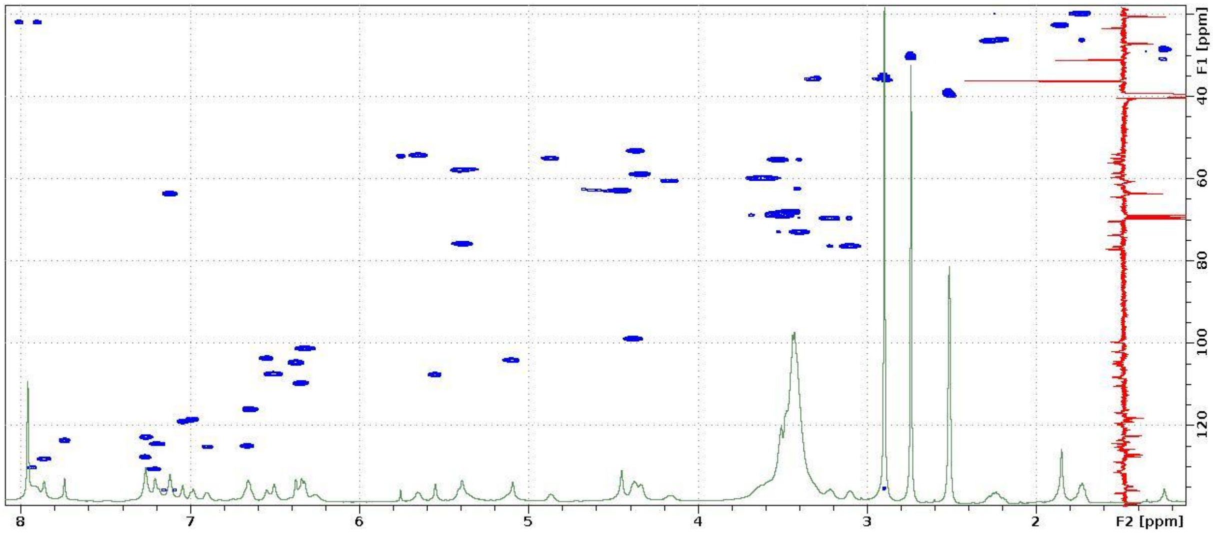
F1 [ppm]
F2 [ppm]

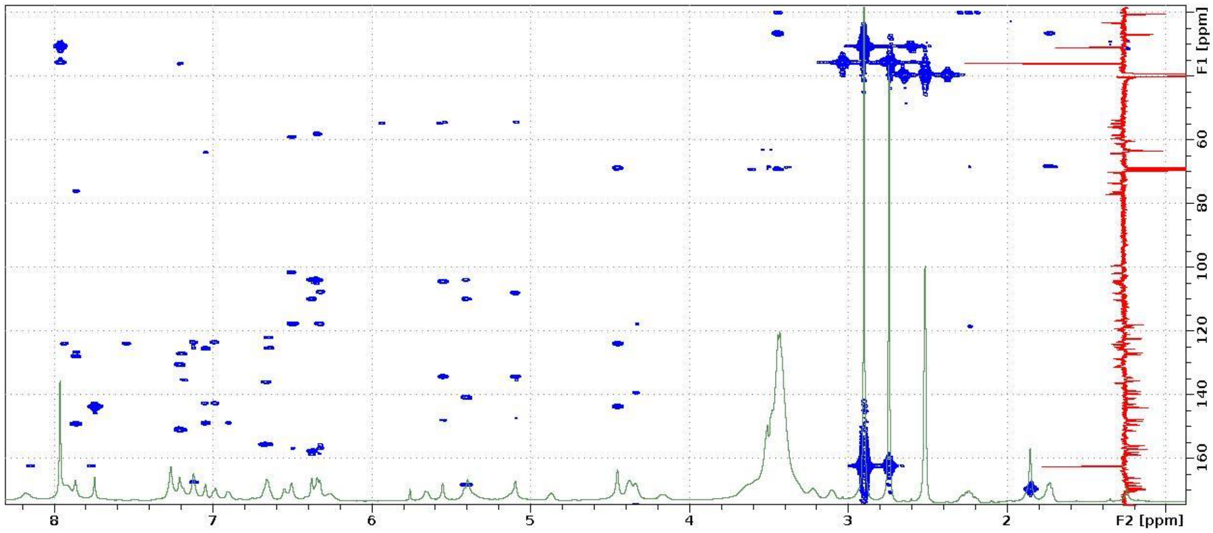
F1 [ppm]
F2 [ppm]

^{19}F, ^{13}C, HSQC and HMBC spectra of compound **16**, (470, 125, 500 MHz) DMSO

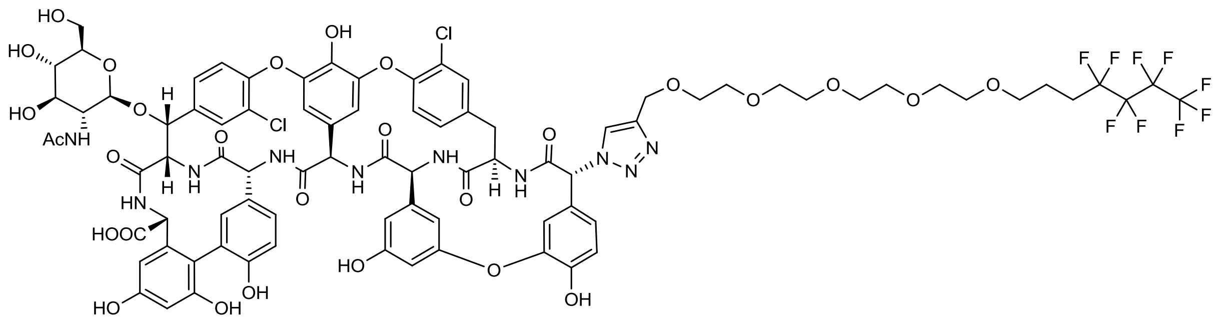

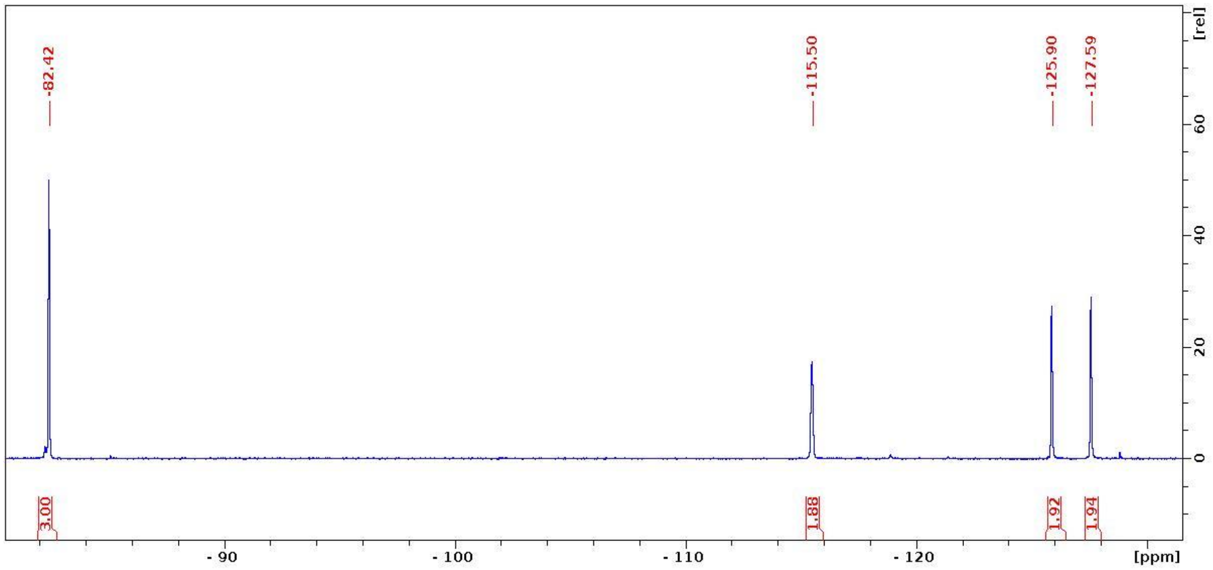

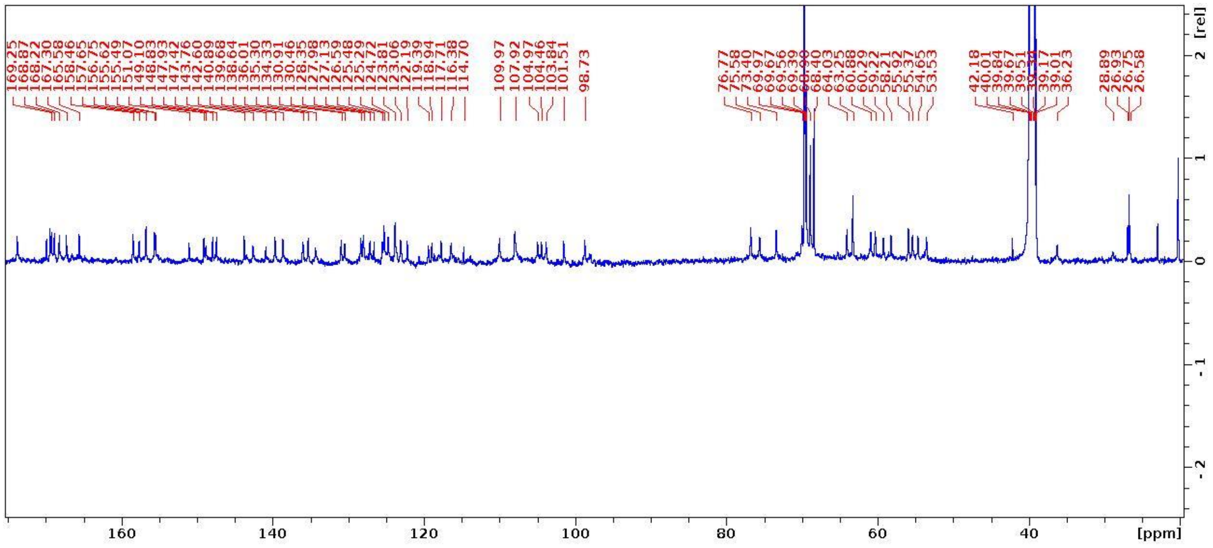

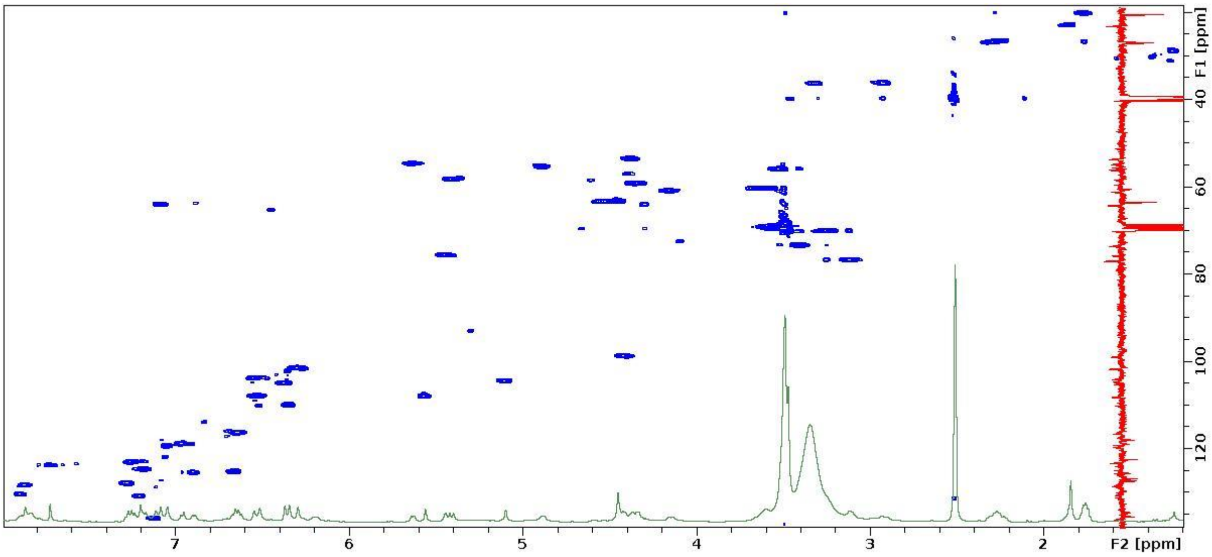
F1 [ppm]
F2 [ppm]

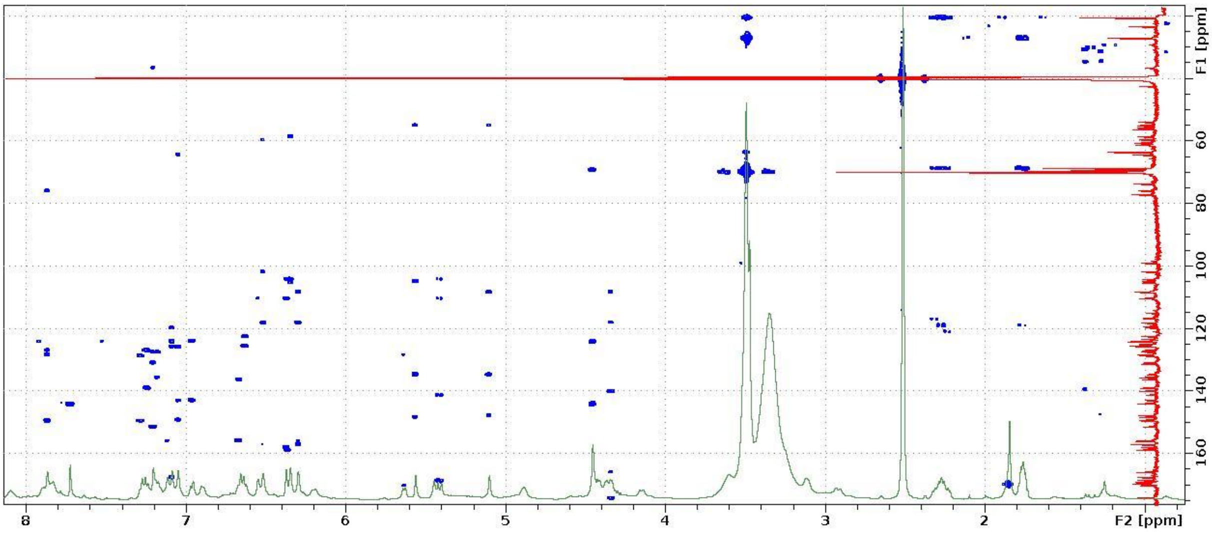
F1 [ppm]
F2 [ppm]

JMOD, COSY, HSQC spectra of compound **17**, (125, 500 MHz) DMSO

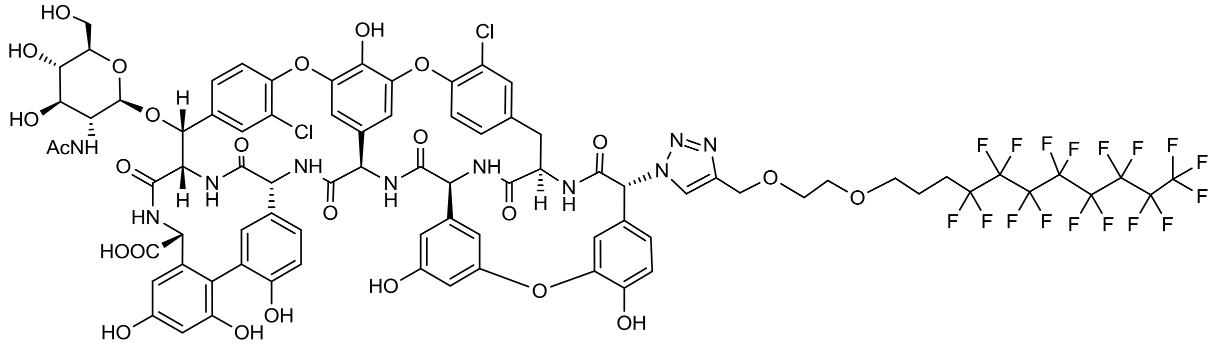

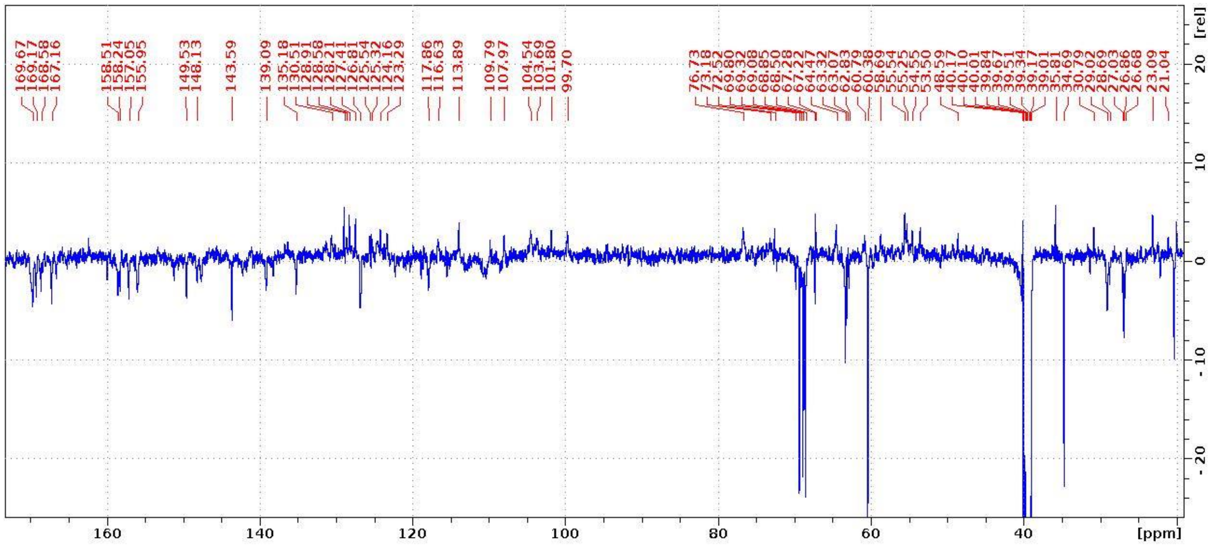

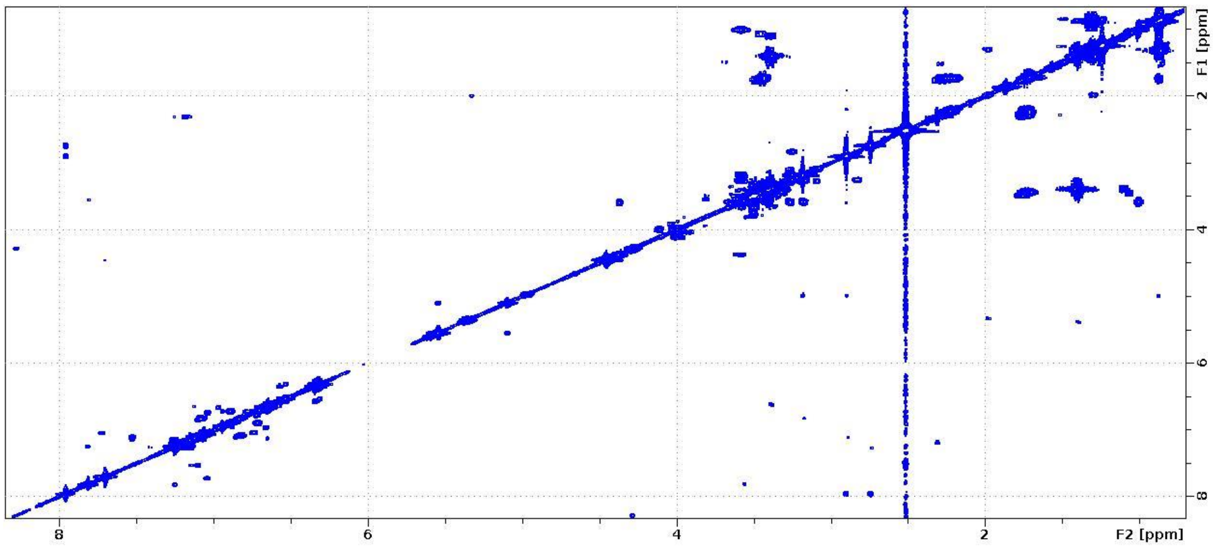

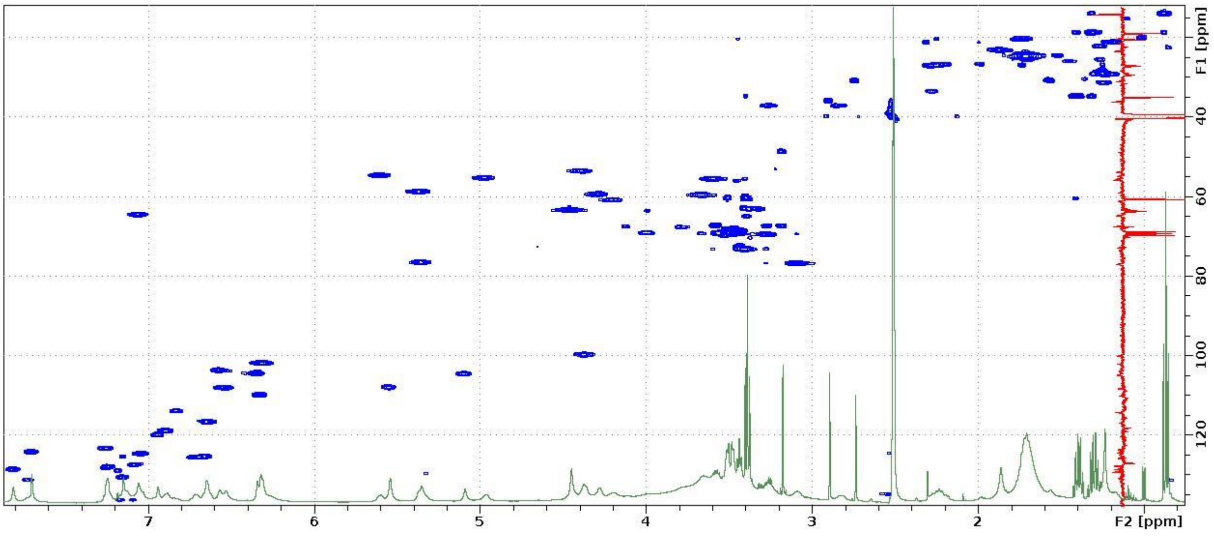

^{13}C, HSQC, HMBC spectra of compound **18**, (125, 500 MHz) DMSO

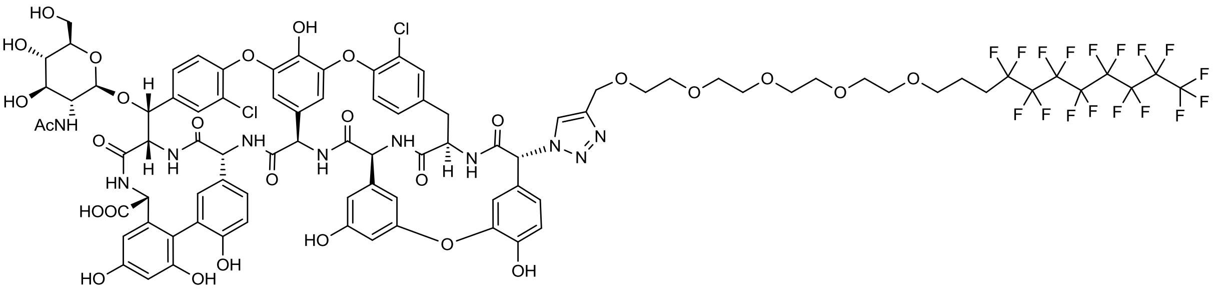

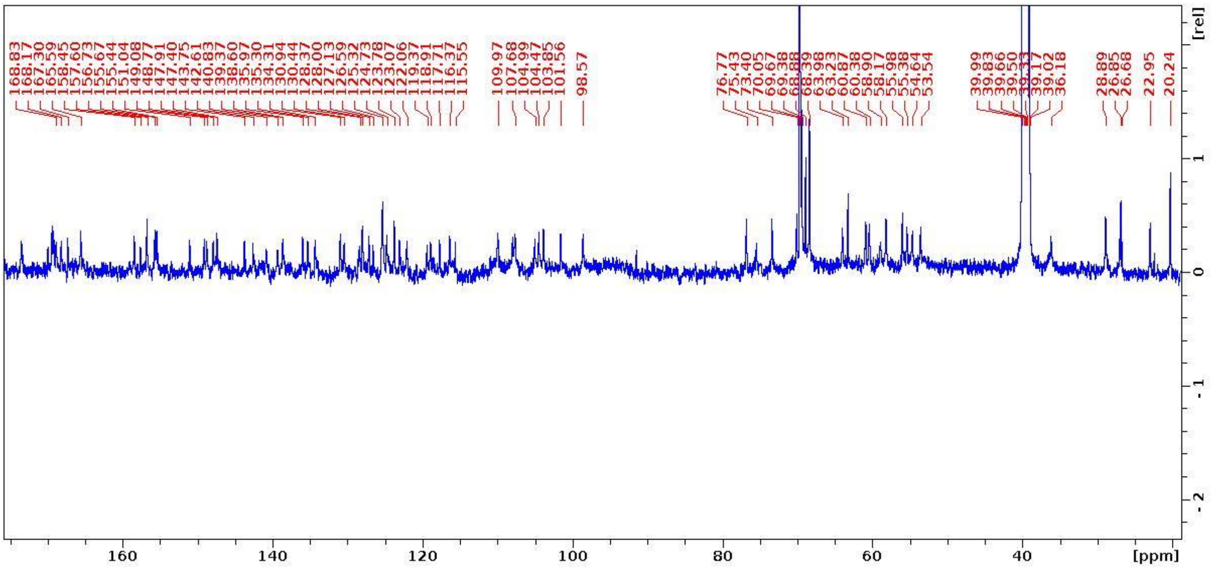

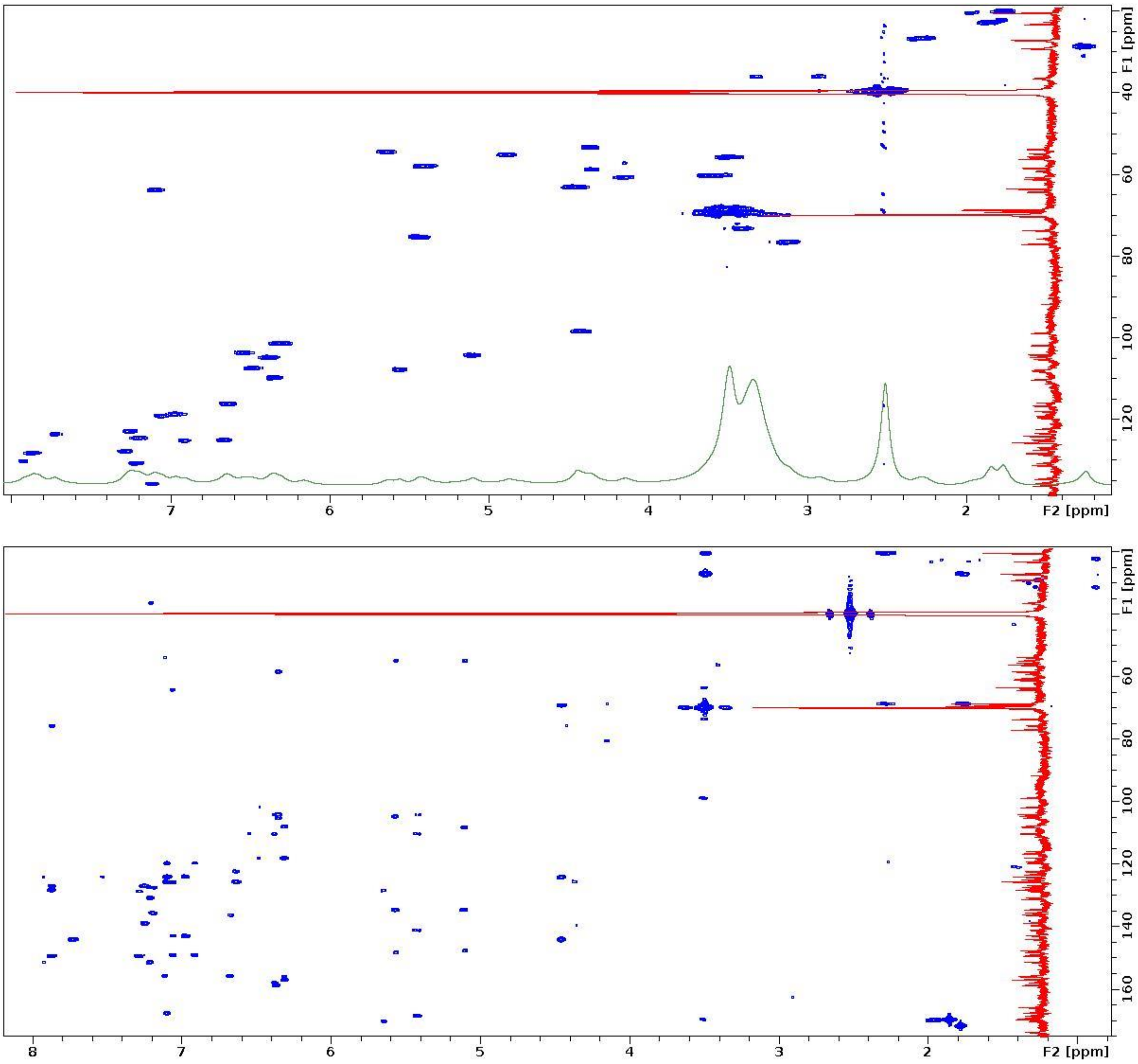

F1 [ppm]
F2 [ppm]
